# Partial-coverage assembly of graphdiyne-derived fragment-protected Cu(I) clusters generates an ordered single-metal site catalyst

**DOI:** 10.1093/nsr/nwaf575

**Published:** 2025-12-15

**Authors:** Shuai Chen, Xi Fan, Shuai Yan, Morgan McKee, Alexandre Terry, Chen Gao, Mahsa Abdolmaleki, Jost Heise, Minmin Chen, Yves Kayser, Serena DeBeer, Jian Zhang, Nikolay Kornienko

**Affiliations:** Institute of Inorganic Chemistry, University of Bonn, Bonn 53121, Germany; Department of Molecular Spectroscopy, Max Planck Institute for Polymer Research, Mainz 55128, Germany; State Key Laboratory of Structural Chemistry, Fujian Institute of Research on the Structure of Matter, Chinese Academy of Sciences, Fuzhou 350002, China; Institute of Inorganic Chemistry, University of Bonn, Bonn 53121, Germany; Institute of Inorganic Chemistry, University of Bonn, Bonn 53121, Germany; Institute of Inorganic Chemistry, University of Bonn, Bonn 53121, Germany; Institute of Inorganic Chemistry, University of Bonn, Bonn 53121, Germany; Institute of Inorganic Chemistry, University of Bonn, Bonn 53121, Germany; Institute of Inorganic Chemistry, University of Bonn, Bonn 53121, Germany; Department of Inorganic Spectroscopy, Max Planck Institute for Chemical Energy Conversion, Mülheim an der Ruhr 45470, Germany; Department of Inorganic Spectroscopy, Max Planck Institute for Chemical Energy Conversion, Mülheim an der Ruhr 45470, Germany; Department of Inorganic Spectroscopy, Max Planck Institute for Chemical Energy Conversion, Mülheim an der Ruhr 45470, Germany; State Key Laboratory of Structural Chemistry, Fujian Institute of Research on the Structure of Matter, Chinese Academy of Sciences, Fuzhou 350002, China; Institute of Inorganic Chemistry, University of Bonn, Bonn 53121, Germany

**Keywords:** Cu cluster, atomic metal cluster, partial-coverage assembly, ordered single-metal sites, cluster catalysis

## Abstract

Isolated single-site catalysts (ISSCs) have emerged as promising materials for energy conversion and storage. However, current approaches for inorganic nanocatalysts are often ineffective in achieving precisely ordered periodic atomic arrangements of active sites, often leading to a random distribution of active-site motifs on an inorganic substrate. In this work, we introduce a novel partial-coverage-assembly strategy, leveraging graphdiyne-derived fragment ligands, to synthesize a unique Cu nanocluster catalyst with an ordered periodic arrangement of isolated single-metal Cu sites [Cu_4_(TFA)_4_(DPBD)_2_, Cu-SMS], while maintaining identical atomicity and a homogeneous coordination microenvironment. This strategic approach significantly enhances the electron transport capability by incorporating graphdiyne-inspired bridging ligands as compared to non-coverage-assembled Cu-MMS (MMS: multiple-metal site). As a result, the Cu-SMS nanocluster catalyst exhibited superior performance in electrocatalytic nitrate reduction to ammonia, achieving a Faradaic efficiency exceeding 99%, surpassing all previously reported atomic precise metal nanocluster catalysts. Through a combination of *in situ* attenuated total reflection surface-enhanced infrared absorption spectroscopy, electrochemical mass spectrometry and density functional theory calculations, we unraveled a detailed mechanistic pathway of nitrate reduction on Cu-SMS, highlighting the role of key intermediates (*NO_2_, *NO, *NHO, *NHOH, *NH_2_OH, *NH_2_) and identifying the rate-determining step. In all, these findings present a novel methodology for synthesizing periodic SMS catalysts, emphasizing the emergent catalytic behaviors of precisely ordered metal clusters in heterogeneous catalysis.

## Teaser

This study presents a novel synthetic strategy, termed partial-coverage-assembly (PCA), which, in combination with graphdiyne fragment ligands, enables the construction of an ordered single-Cu-site catalyst (Cu-SMS). The resulting catalyst exhibits enhanced electron transport capability and delivers superior performance in the electrochemical nitrate reduction to ammonia.

## INTRODUCTION

**Figure 1.**
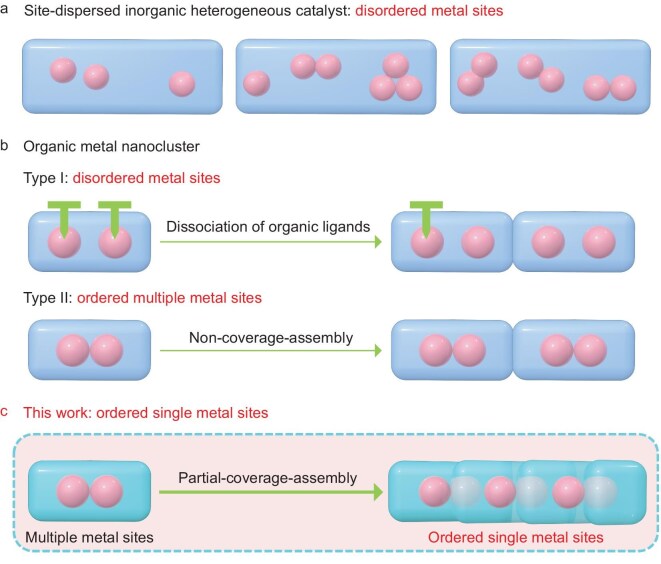
Schematic comparison of disordered and ordered metal sites. (a) Conventional site-dispersed inorganic heterogeneous catalyst featuring disordered metal sites. (b) Organic-ligands-dissociation strategy for generating disordered metal sites (type I), and non-coverage-assembly approach for forming ordered multiple metal sites (type II) in organic metal nanoclusters. (c) Partial-coverage-assembly strategy for constructing homogeneous and ordered single-metal sites.

Transforming abundant, non-fossil feedstocks, such as nitrates, water and carbon dioxide into valuable fuels and chemicals presents a promising approach to mitigating global warming and effectively storing renewable energy [1‒3]. Within this context, renewable electrosynthetic pathways have gained significant attention, with isolated single-site catalysts (ISSCs) standing out as particularly promising [4‒6]. Unlike conventional heterogeneous catalysts, by anchoring individual catalytic centers on well-defined supports at atomic or molecular scales, ISSCs enable precise tuning of catalytic activity and selectivity by virtue of having well-defined active-site motifs [7‒10]. A challenge in this regard is that the characterization of ISSCs extensively relies on advanced microscopy and synchrotron radiation techniques [11‒14]. Furthermore, anchoring sites are often sparse and disordered, resulting in inconsistent distribution of single-site catalysts across inorganic solid supports and thus a heterogeneity of active site distribution and catalytic microenvironments (Fig. 1a) [15]. Thus, achieving uniformly ordered, periodic atomic arrangements of active sites with near-perfect atomic efficiency and tunable electronic configurations is crucial. Such advancements would significantly enhance the rational design of catalysts, provide deeper insights into catalytic mechanisms, and establish robust structure–performance relationships within the catalyst microenvironment. Further, emergent behaviour like enhanced electronic conductivity may arise from a periodic ordering as π-conjugated ligands are consistently positioned next to each other.

Cu-based catalysts (e.g. single-atom catalysts, molecular catalysts and nanostructured catalysts), have shown strong potential for key electrochemical reactions such as nitrate reduction (NO_3_RR), owing to their diverse oxidation states, moderate carbon-binding strength and cost-effectiveness [14]. However, these catalysts are often prone to structural reconstruction during the reaction, complicating the elucidation of a clear structure–activity relationship. As molecular models of metal nanoparticles, atomically precise metal nanoclusters (MNCs) [16‒20], especially Cu-based nanoclusters, are particularly appealing platforms for constructing uniformly distributed and stable single-metal active sites with atomic precision [21‒25]. However, under operating conditions, exposing these catalytic sites often necessitates partial ligand dissociation from the nanoclusters, which might reduce the cluster stability and lead to a disordered distribution of catalytic sites (Fig. 1b, type I) [26]. While certain ligands, such as pyrazole, can directly expose metal sites through their unique planar coordination geometry, this approach typically results in multi-site exposure. Nonetheless, the inherent diversity of multiple sites complicates the precise control of catalytic mechanisms (Fig. 1b, type II) [27]. Given the advantages of single-site catalysts, these challenges can be addressed by anchoring single active sites onto Cu nanoclusters. However, research on single catalytic sites in MNCs is still in its early stages, and strategies for controllably exposing single sites on Cu nanoclusters remain largely unexplored.

Graphdiyne, an emerging 2D carbon allotrope consisting of sp- and sp^2^-hybridized carbon atoms, has attracted considerable attention as a new class of carbon materials [28,29]. Its unique uniform pores, highly π-conjugated structure, tunable bandgaps and high charge-carrier mobility endow graphdiyne with broad potential for applications in electrocatalysis, energy storage, gas separation, sensing and optoelectronics [30‒33].

In heterogeneous catalysis, clusters often interact through stacking, forming crystalline structures [21]. The stacking mode between clusters is a critical factor in determining the nature of active sites from a catalytic perspective. However, previous studies have predominantly focused on isolated clusters, often overlooking their aggregation and consequent emergent properties [22,23]. Graphdiyne offers an extended π-conjugated framework, a strong coordination environment for Cu, and efficient pathways for electron transport [34]. In this work, we utilized graphdiyne-derived fragment ligands, specifically 1,4-diphenylbutadiyne (DPBD), to synthesize a novel Cu nanocluster [Cu_4_(TFA)_4_(DPBD)_2_], referred to as Cu-SMS, where SMS denotes single metal site and TFA is trifluoroacetic acid. Through a partial-coverage assembly, we enabled a controlled interaction between the Cu clusters, resulting in homogeneous and ordered single-metal catalytic sites (Fig. 1c). Notably, Cu-SMS represents the first Cu nanocluster stabilized by graphdiyne-derived fragment ligands. Those graphdiyne-inspired bridging ligands enhance intramolecular electron delocalization within each cluster, while partial-surface-coverage assembly minimizes inter-cluster energy barriers. Single-crystal conductivity measurements revealed that Cu-SMS exhibits over 20-fold higher conductivity than its multi-site counterpart, Cu-MMS (MMS = multiple metal site, non-π-mode ligands, non-coverage assembly). Remarkably, Cu-SMS exhibits superior performance in the electrochemical NO_3_RR, attaining exceptionally a Faradaic efficiency approaching 99% at a current density of 40 mA cm^−2^, while maintaining long-term catalytic stability for over 7 h. Under operating conditions, the single Cu catalytic sites exhibit excellent stability, as confirmed through extended X-ray absorption fine structure (EXAFS) spectroscopy, X-ray photoemission spectroscopy (XPS) and powder X-ray diffraction (PXRD) analysis. In parallel, to elucidate the mechanistic pathways of NO_3_RR activity on Cu-SMS, we conducted *in situ* attenuated total reflection surface-enhanced infrared absorption spectroscopy (ATR-SEIRAS), electrochemical mass spectrometry (ECMS) and density functional theory (DFT) calculations. These studies indicate that NO_3_^−^ is first reduced to *NO_2_ on the Cu-SMS surface, followed by a cascade of intermediates (*NO, *NHO, *NHOH, *NH_2_OH and *NH_2_) leading to NH_3_ formation. Together, these findings provide a deeper insight into the structure–activity relationship of Cu-SMS and provide insights into the rational design of ordered molecular electrocatalytic systems.

## RESULTS AND DISCUSSION

### Synthesis and characterization of Cu-SMS and Cu-MMS molecular nanocluster catalysts

**Figure 2.**
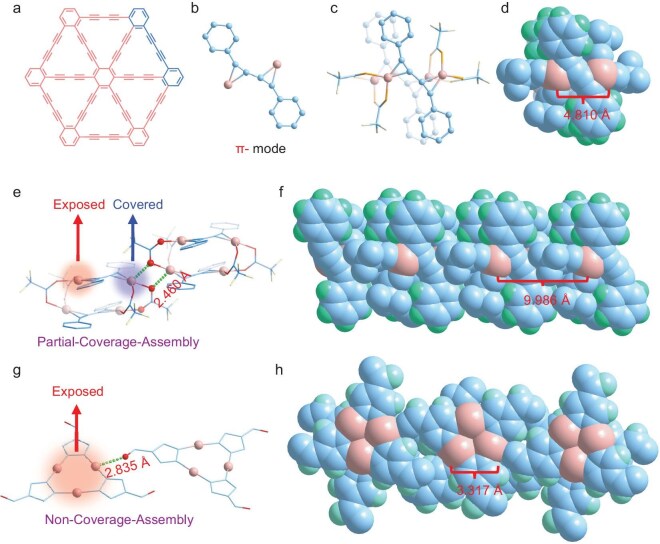
Crystal structures of Cu-SMS and Cu-MMS. (a) Schematic structure of graphynes, highlighting the graphdiyne fragment. (b) Coordination modes of graphdiyne-derived fragment ligands with Cu atoms in π mode. (c) Molecular structure of Cu-SMS (H atoms omitted for clarity). (d) Space-filling model of the Cu-SMS structure. (e) Partial-coverage assembly in Cu-SMS, where single Cu atoms are exposed on the surface. (f) Packing structure of single Cu sites in Cu-SMS, highlighting the ordered single-metal sites. (g) Non-coverage assembly in Cu-MMS, exposing multiple Cu atoms on the surface. (h) Packing structure of multiple sites in Cu-MMS.

An ideal catalyst maximizes the number of exposed active sites and optimizes intrinsic activity at each site to enhance heterogeneous reactions. Typically, in polynuclear Cu clusters, alkynyl ligands act as terminal ligands via σ-coordination modes or coupled with π-coordination modes (Fig. S1), effectively blocking potential open Cu sites. Previous reports have indicated that Cu atoms can coordinate with alkynes in graphdiyne (Fig. 2a) through a π-coordination mode [24]. Inspired by these studies, we introduce graphdiyne-derived fragment ligands that have coupled alkyne groups and lose their σ-coordinating capability (Fig. 2b), thereby preventing them from functioning as terminal ligands. This unique coordination mode facilitates the formation of open catalytic sites, although the ligand has not yet been used for protecting Cu clusters.

Using a one-pot solvothermal method, Cu_2_O, TFA and DPBD reacted in acetonitrile (CH_3_CN) at 85°C for 1 day, yielding yellow block-shaped crystals of Cu-SMS. Single-crystal X-ray diffraction analysis revealed that Cu-SMS features a small quadrilateral Cu-SMS core, stabilized by four TFA and two DPBD ligands (Fig. 2c). As no reductant was added during the reaction, the cluster contains no hydrides (H^−^), indicating that Cu in Cu-SMS exists in a Cu^+^ oxidation state, with no free valence electrons. Two Cu atoms are π-coordinated on each side of the R–C≡C–C≡C–R unit, with a distance of 4.810 Å between them (Fig. 2d). These alkynyl Cu_2_ units are further stacked via four carboxylic acids, forming the final Cu-SMS structure. Each Cu atom only coordinates with one C≡C and two oxygen atoms, with these five atoms nearly lying in a plane arrangement, allowing each Cu atom in the cluster to have active sites.

In heterogeneous catalysis, clusters typically interact through stacking to form crystalline structures, making the stacking mode critical when considering active sites. Interestingly, in the Cu-SMS cluster, one Cu site exhibits quasi-coordination with the oxygen from the TFA on a neighboring Cu-SMS (2.460 Å), resulting in the sealing off of this site after stacking and leaving only one active site exposed (Fig. 2e). We refer to this packing behavior in the cluster as ‘partial-coverage assembly (PCA)’. After stacking through PCA, the distance between the remaining active Cu sites extends to 9.986 Å (Fig. 2f), effectively transforming the stacked Cu-SMS into a single-site catalyst. To further confirm the single-metal sites on the crystal surface, we analyzed the crystal structure of Cu-SMS along all three axes (Fig. S4a, b and c). Along the *a*-axis (Fig. S4a), the structure exclusively exposes spatially isolated single Cu sites, while along the *c*-axis, no significant exposure of active sites is observed (Fig. S4b). In contrast, viewing along the *b*-axis reveals that the stacking of clusters can locally generate incompletely isolated boundaries, where dual-site exposure may occur (Fig. S4c). However, these edge-boundary regions constitute a negligible fraction of the total crystal surface. Therefore, on a macroscopic scale, the fraction of cluster molecules on the crystal edge-boundary region is virtually negligible. The vast majority of exposed sites in Cu-SMS are indeed single sites, which supports our classification of Cu-SMS as a single-site catalyst.

To compare, we synthesized another atomically precise Cu nanocluster with well-defined catalytic sites, Cu-MMS, through a non-coverage-assembly approach [25]. Similarly to Cu-SMS, Cu-MMS contains multiple metal sites within its cluster unit. However, the π-mode graphdiyne-derived fragment bridging in Cu-SMS drives partial-coverage assembly and thereby creates a single-metal site, whereas the absence of this motif in Cu-MMS results in non-coverage assembly and multiple-metal sites. Structural analysis of Cu-MMS revealed a Cu_3_ kernel coplanarity safeguarded by three 4-formylpyrazole ligands. Analogous to Cu-SMS, the oxygen atoms on the carbonyl group of the Cu-MMS cluster establish quasi-coordination with the Cu atoms on adjacent clusters, featuring a Cu‒O distance of 2.835 Å (Fig. 2g). However, as the O atoms remain nearly coplanar with the Cu atoms, this quasi-coordination does not obstruct the unsaturated sites on the upper ends of the Cu atoms, demonstrating the ‘non-coverage-assembly’ mode in Cu-MMS. As a result, multiple-metal sites within the outermost clusters of the crystal are preserved, with an average distance between Cu atoms in Cu-MMS being 3.317 Å (Fig. 2h), thereby rendering Cu-MMS a multi-site catalyst.

### The conductivity of Cu-SMS and Cu-MMS single crystals

**Figure 3.**
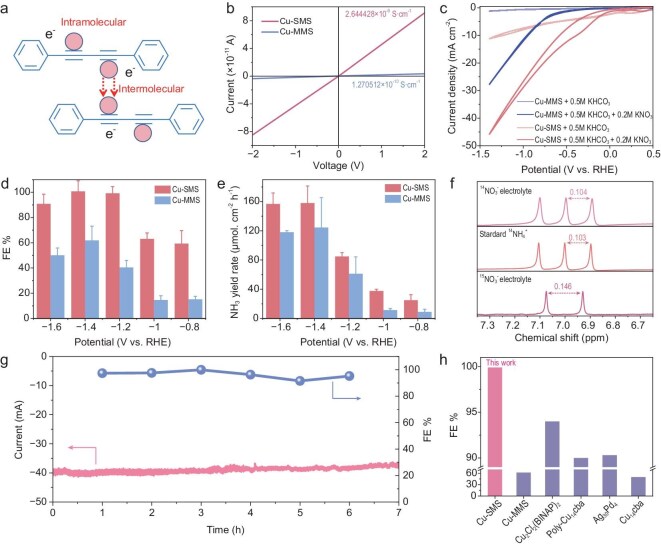
Electrochemical NO_3_RR performances of Cu-SMS and Cu-MMS molecular nanocluster catalysts. (a) Intra- and inter-molecular electron transfer in the Cu-SMS. (b) Single-crystal conductivity measurements of Cu-SMS and Cu-MMS. (c) CV curves of Cu-SMS and Cu-MMS in the absence and presence of KNO_3_ in a neutral pH environment. (d) Faradaic efficiencies of Cu-SMS and Cu-MMS at varying applied potentials. (e) NH_3_ yield rate of Cu-SMS and Cu-MMS as a function of applied potentials. (f) ^1^H NMR spectra of standard sample (^14^NH_4_)_2_SO_4_ and 0.5 M KHCO_3_ electrolyte after NO_3_RR at −1.2 V using ^14^NO_3_^−^ and ^15^NO_3_^−^ as N sources, respectively. (g) Long-time stability test of Cu-SMS at a current density of 40 mA cm^−2^. The left axis represents the current density, while the right axis shows the Faradaic efficiencies of NO_3_RR products. (h) Comparison of the Faradaic efficiencies of NO_3_RR among representative organic metal nanocluster materials.

The electrical conductivity of electrocatalysts plays a pivotal role in their performance, given the requirement for electron transfer between the electrode, catalyst film and reactants. For nanocluster catalysts, both the intrinsic molecular structure of individual clusters and the inter-cluster interactions critically influence their conductive properties. Graphdiyne, characterized by alternating benzene rings (sp^2^-hybridized) and diacetylene linkages (sp-hybridized), forms a 2D planar conjugated network. This unique architecture endows graphdiyne with exceptional carrier mobility and electrical conductivity surpassing that of doped silicon. As a structural derivative of graphdiyne, the DPBD fragment is anticipated to enhance intramolecular electron transport within cluster cores by leveraging its conjugated backbone. When atomically precise metal nanoclusters are utilized in heterogeneous catalysis, weak intermolecular interactions (e.g. van der Waals forces and hydrogen bonding) typically dominate inter-cluster connections. These interactions often hinder efficient electron transfer between adjacent clusters, consequently limiting catalytic efficiency. Notably, Cu-SMS employs a partial-coverage-assembly strategy with dual quasi-coordination bonds. This approach that leverages the graphdiyne fragment may synergistically enhance charge transport through two mechanisms: (i) graphdiyne-inspired bridging ligands optimize intramolecular electron migration within individual clusters; and (ii) partial-surface-coverage assembly minimizes inter-cluster energy barriers (Fig. 3a). Experimental validation through single-crystal conductivity measurements revealed striking performance differences: Cu-SMS exhibits a conductivity exceeding that of Cu-MMS (monomeric metal cluster system) by over 20-fold (Fig. 3b and Fig. S5). The improved charge transport dynamics directly correlate with enhanced catalytic activity in redox-mediated reactions.

Importantly, this partial-coverage-assembly strategy has the potential to be generalized to other π-conjugated ligands and diverse metal centers. Noble metals (e.g. Ag, Au) could benefit from stabilized single-metal sites with enhanced charge mobility, while earth-abundant transition metals (e.g. Fe, Co, Ni) could be tailored for energy-relevant electrocatalytic reactions such as CO_2_ reduction, oxygen reduction or nitrogen fixation. Overall, this strategy, integrating conjugated ligands with ordered molecular packing, provides a broadly applicable design principle for engineering electronically conductive, atomically precise catalysts.

### Investigation of NO_3_^−^ electroreduction for Cu-SMS and Cu-MMS molecular nanocluster catalysts

As an initial probe of potential differences in their reactivity, the NO_3_RR activity of Cu-SMS and Cu-MMS molecular nanocluster catalysts was evaluated through electrochemical measurements. First, cyclic voltammetry (CV) at a scan rate of 20 mV s^−1^ was performed for both catalysts. As shown in Fig. 3c, the presence of 0.2 M NO_3_^−^ resulted in an enhanced current density compared to the absence of NO_3_^−^, indicating the high potential of molecular catalysts for NO_3_RR in KHCO_3_ and KNO_3_ electrolytes of neutral pH. The CV results demonstrated that Cu-SMS exhibited a higher current density across the tested potential range, highlighting its superior catalytic activity compared to Cu-MMS, when using an equivalent catalyst loading (same quantity of Cu sites) on the electrode.

Subsequently, the NO_3_RR activity was further investigated using chronoamperometry in a 0.5 M KHCO_3_ and 0.2 M KNO_3_ electrolyte, at applied potentials ranging from −0.8 to −1.6 V vs. the reversible hydrogen electrode (RHE) (Fig. 3d). Liquid products were quantified using both proton nuclear magnetic resonance (^1^H NMR) spectroscopy and ultraviolet-visible (UV–vis) spectrophotometry, based on standard calibration curves (Figs S11 and S12). For ^1^H NMR, ammonia quantification was conducted using maleic acid as an external standard. As shown in Fig. 3d, the NH_3_ Faradaic efficiency displayed a volcano-shaped trend with increasing applied potentials for Cu-SMS. The highest NH_3_ Faradaic efficiency was observed at −1.4 V vs. RHE, with Cu-SMS achieving over 99% and Cu-MMS reaching 56%. Figure3e shows that the NH_3_ yield rate increased progressively as the applied potential became more negative for both catalysts. Cu-SMS consistently outperformed Cu-MMS across the tested potential range, and Cu-MMS produced more NO_2_^−^ instead and was less effective in carrying out the entire 8*e*^−^ reduction process. Despite the higher active site density per unit area in the multi-site catalyst (Cu-MMS), the single-site catalyst (Cu-SMS) demonstrated superior yield. This apparent discrepancy can be attributed to two factors: (i) the significantly higher Faradaic efficiency of Cu-SMS (>99% vs. 56% for Cu-MMS at optimal potential); and (ii) enhanced charge transfer efficiency through optimized electronic conductivity in the single-site configuration.

To confirm the reliability of these results, a series of control experiments were conducted. As shown in Fig. S13, no ammonia was detected in the absence of NO_3_^−^. Similarly, bare carbon paper without loaded catalysts produced no ammonia, confirming that the observed NH_3_ primarily originated from the loaded catalysts in the presence of NO_3_^−^ under the applied potentials. The origin of the produced ammonia was verified by ¹H NMR. Figure3f shows characteristic double peaks at 7.07 and 6.93 ppm when K^15^NO_3_ was used as a nitrogen source, corresponding to ^15^NH_4_^+^. Triple peaks at 7.10, 7.00 and 6.89 ppm were observed with K^14^NO_3_, indicating the formation of ^14^NH_4_^+^ and confirming that the ammonia originated from the NO_3_^−^ in the electrolyte. The stability of Cu-SMS was evaluated through 7 h of continuous electrolysis (Fig. 3g). The stable NH_3_ Faradaic efficiency and current density over this period demonstrated the excellent durability of Cu-SMS for NO_3_RR within this timeframe. In comparison, the Cu-SMS outperformed Cu-MMS in nitrate-to-ammonia conversion, achieving a Faradaic efficiency exceeding 99%—surpassing previously reported atomically precise metal cluster catalysts (Fig. 3h) [26,27,35] and other Cu-based catalysts (Table S4) [36‒47].

### Investigation of oxidation state and structure stability during the NO_3_RR

**Figure 4.**
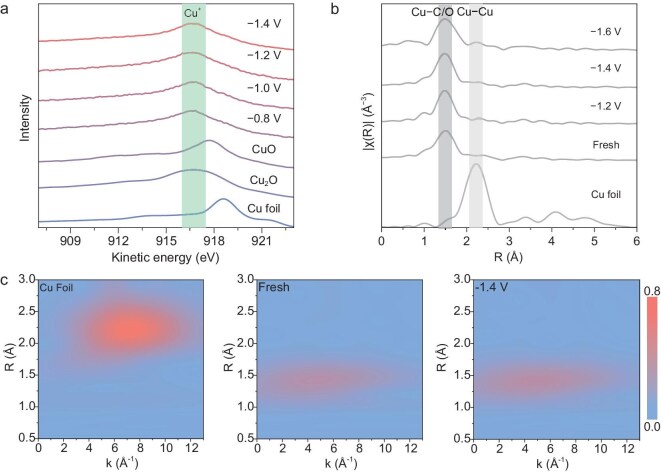
Stability of Cu-SMS. (a) Cu LMM Auger spectra were obtained at potentials from −0.8 V to −1.4 V vs. RHE, along with reference samples (Cu foil, Cu_2_O, CuO). (b) Cu K-edge FT-EXAFS spectra were recorded at various applied potentials, ranging from fresh to −1.6 V vs. RHE, compared with the reference sample (Cu foil). (c) WT EXAFS spectra of the corresponding samples.

The structural and chemical state changes of Cu-SMS were investigated using *ex situ* XPS and EXAFS following electrolysis at various potentials. XPS analysis probed the oxidation states (Cu^0^, Cu^+^ or Cu^2+^) of Cu elements in Cu-SMS. The full XPS survey spectrum reveals the coexistence of Cu, F, O and C in the synthesized Cu-SMS (Figs S14–S17). Notably, across the potential range of −0.8 to − 1.4 V vs. RHE, Cu L-shell to M-shell to M-shell (LMM) Auger electron spectroscopy confirmed the surface composition to be predominantly Cu⁺, as evidenced by the presence of the characteristic Cu⁺ peak at 916.8 eV (Fig. 4a, Figs S18 and S19). This indicates that the Cu on the surface of Cu-SMS is primarily in the Cu⁺ oxidation state rather than Cu^0^ or Cu^2+^.

Upon applying potential ranging from 0 to −1.6 V vs. RHE, the k^2^-weighted χ(k) indicated that the local coordination environment of the Cu-SMS samples remained similar under conditions ranging from the fresh state to −1.6 V vs. RHE (Fig. S20). Moreover, the Fourier-transforms of EXAFS (FT-EXAFS) spectra (Fig. 4b) showed no clear peak alignment with the reference Cu foil sample, further supporting the absence of metallic Cu formation and the preservation of the Cu-SMS structure. These findings, consistent with the wavelet transform (WT) of Cu K-edge EXAFS results (Fig. 4c), suggest that Cu-SMS maintains its fundamental structural integrity under the tested electrochemical conditions. Additionally, PXRD analyses indicate that the Cu-SMS crystal retains its crystallinity in the neutral environment of 0.5 M KHCO_3_ and 0.2 M KNO_3_ after catalytic testing (Fig. S21). Furthermore, scanning electron microscopy (SEM) and transmission electron microscopy (TEM) analyses were conducted to examine the morphology features of the catalysts (Figs S22–S28). The morphology of Cu-SMS remained unchanged, whereas Cu-MMS exhibited slight morphological variations.

### Reaction mechanism via *in situ* analysis and DFT calculations

**Figure 5.**
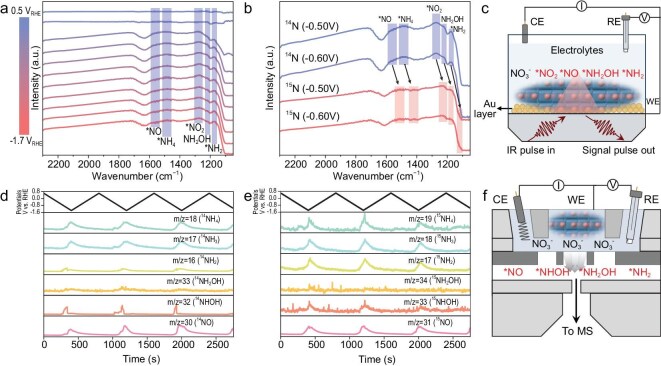
Electrochemical *in situ* analysis of NO_3_RR over Cu-SMS. (a) Potential-dependent *in situ* ATR-SEIRAS spectra. (b) Comparison of isotope-labeled (K^15^NO_3_) and normal nitrogen source (K^14^NO_3_) in *in situ* ATR-SEIRAS analysis. (c) Schematic illustration of the *in situ* ATR-SEIRAS measurement setup and detected intermediates. (d) Potential-dependent *in situ* ECMS spectra. (e) Potential-dependent isotope-labeled *in situ* ECMS analysis using K^15^NO_3_. (f) Schematic illustration of the *in situ* ECMS measurement setup and detected intermediates. CE: counter electrode, RE: reference electrode, WE: working Electrode, IR: infrared, MS: mass spectroscopy.

The catalytic pathways and underlying mechanisms for NO_3_RR over Cu-SMS were investigated using a combination of *in situ* electrochemical techniques, including ATR-SEIRAS and ECMS. These techniques allowed for the detection of intermediate species and provided detailed insights into the NO_3_RR reaction pathways and the role of Cu-SMS molecular nanocluster catalysts. *In situ* ATR-SEIRAS monitored the formation of intermediates nearby and adsorbed on the electrode surface. Figure 5a, and Figs S29 and S30 show potential-dependent ATR-SEIRAS for NO_3_RR over Cu-SMS, recorded from 0.50 to −1.70 V vs. RHE. To validate and support our findings, we referenced relevant literature and provided detailed attributions in the Supplementary data, as summarized in Table S2. A positive band at 1274 cm^−1^, associated with *NO_2_ formation [48‒50], intensifies as the potential becomes more negative. Another band at 1565 cm^−1^, corresponding to the formation of *NO [46], is also observed. At −0.50 V vs. RHE, a weak band at 1221 cm^−1^, attributed to NH_2_OH [48], appears, suggesting that part of the reaction follows the pathway: NO_3_ → *NO_2_ → *NO → *NH_2_OH → *NH_2_ → *NH_4_^+^ (Fig. 5c). Additionally, a characteristic peak at 1178 cm^−1^, corresponding to *NH_2_, is detected [46,49,51,52]. These results suggest that NO_3_^−^ is initially reduced to *NO_2_ on Cu-SMS, and further reduction steps lead to NH_3_. As shown in Fig. 5b, electrochemical isotope-labeling *in situ* ATR-SEIRAS experiments further confirmed that the reaction intermediates contained nitrogen. When using K^14^NO_3_ as the nitrogen source, characteristic peaks for ^14^*NO, ^14^NH_4_^+, 14^*NO_2_, ^14^*NH_2_OH and ^14^*NH_2_ (1565, 1482, 1274, 1221 and 1178 cm^−1^, respectively) were observed. When K^15^NO_3_ was used, these peaks shifted to lower wavenumbers due to the isotope effect: ^15^*NO, ^15^NH_4_^+, 15^*NO_2_, ^15^*NH_2_OH and ^15^*NH_2_ appeared at 1513, 1417, 1230, 1180 and 1103 cm^−1^, respectively.

Complementarily, online ECMS measurements during NO_3_RR over Cu-SMS (Fig. 5d) further validated the assigned identities of reaction intermediates. The mass/charge (*m*/*z*) signals of 18, 17 and 16, corresponding to NH_4_^+^ and its fragments, were detected over three CV cycle measurements (20 mV s⁻^1^, 0.6 to −1.4 V vs. RHE). Further, volatile gaseous intermediates of *^14^NO (*m*/*z* = 30), *^14^NHOH (*m*/*z* = 32), *^14^NH_2_OH (*m*/*z* = 33), *^14^NH_2_ (*m*/*z* = 16), *^14^NH_3_ (*m*/*z* = 17) and *^14^NH_4_^+^ (*m*/*z* = 18) were observed [49,53], confirming their roles as intermediates in the reaction pathway. Isotope-labeling *in situ* ECMS further corroborated these assignments (Fig. 5e). Signals for ^15^*NO, ^15^*NHOH, ^15^*NH_2_OH, ^15^*NH_2_, ^15^*NH_3_ and ^15^*NH_4_^+^ in the K^15^NO_3_ experiment aligned with their counterparts from the K^14^NO_3_ experiment (Fig. 5d), reinforcing the identification of the proposed intermediates (Fig. 5f). The signal of ^15^*NH_2_OH (*m*/*z* = 34) appears significantly weaker than other dominant intermediates, which can be attributed to the short-lived nature of NH_2_OH during our reaction [54,55].

The combined data from ATR-SEIRAS and ECMS indicate that NO_3_^−^ is first reduced to *NO_2_ on the Cu-SMS catalyst, followed by successive steps involving intermediates (*NO, *NHOH, *NH_2_OH and *NH_2_) that lead to NH_3_ formation. These results offer comprehensive insights into the NO_3_RR reaction mechanism on Cu-SMS, suggesting that the reaction follows the pathway: NO_3_ → *NO_2_ → *NO → *NHOH → *NH_2_OH → *NH_2_ → *NH_3_.

**Figure 6.**
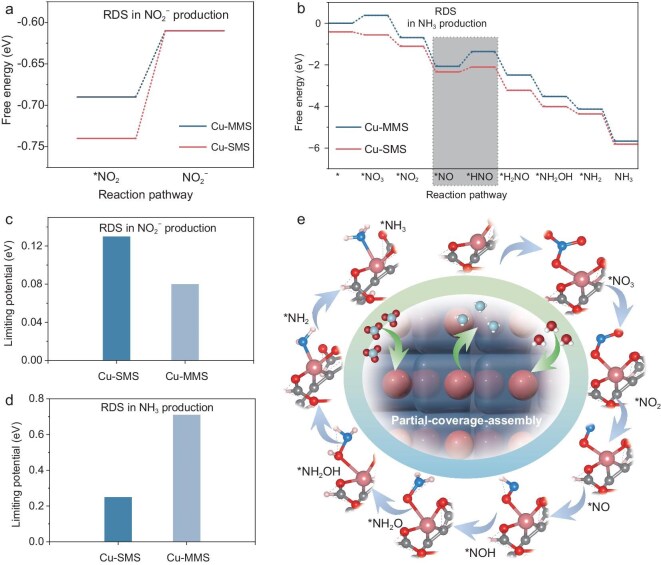
Proposed reaction pathway and DFT calculations for NO_3_RR. (a) Gibbs Free energy diagrams for NO_3_RR to NO_2_^−^ on the surfaces of Cu-SMS and Cu-MMS. (b) The reaction Gibbs free energy trend of NO_3_RR on Cu-SMS and Cu-MMS. (c) Comparison of the energy barriers for the rate-determining step in NO_2_^−^ production on Cu-SMS and Cu-MMS. (d) Comparison of the energy barriers for the rate-determining step in NH_3_ production on Cu-SMS and Cu-MMS. (e) Comprehensive reaction pathways for NO_3_RR on Cu-SMS, integrating insights from *in situ* ATR-SEIRAS, ECMS and DFT calculations.

To further elucidate the NO_3_RR reaction mechanism and free-energy landscape using insights from *in situ* spectroscopy for Cu-SMS and Cu-MMS molecular nanocluster catalysts, DFT calculations were performed (Fig. 6). Detailed information on the DFT calculations is provided in the Experimental section of the Supplementary data. As a starting point, we used the *in situ* spectroscopic analysis to identify key intermediate species, including *NO_2_, *NO, *NHOH, *NH_2_OH and *NH_2_ (Fig. 5). Figure6a and b are to be considered in the DFT Gibbs free energy calculations. The results from the DFT calculations confirmed the presence of intermediate species such as *NO_2_, *NO, *NHO, *NHOH, *NH_2_OH and *NH_2_, consistent with *in situ* spectroscopic findings, except for *NHO, which may have been too short-lived to be detected by the measurement. In the initial reaction steps, the Gibbs free energy values for Cu-MMS (0.38 eV) and Cu-SMS (−0.16 eV) upon initial O atom coordination indicate that Cu-SMS provides a more favorable environment for NO_3_RR. Moreover, Cu-SMS consistently exhibited lower Gibbs free energy values for all intermediates compared to Cu-MMS, illustrating an easier catalytic pathway. The rate-determining step (RDS) of NH_3_ production was identified as the transition from NO* to HNO*. For the byproduct NO_2_^−^, the energy barrier for the RDS on Cu-SMS is higher (0.13 eV) than that on Cu-MMS (0.08 eV), as illustrated in Fig. 6a and c. This indicates that Cu-SMS exhibits superior catalytic selectivity by suppressing undesired by-product formation. In contrast, as shown in Fig. 6b and d, for NH_3_ production, the energy barrier for this RDS on Cu-SMS is significantly lower (0.25 eV) than that on Cu-MMS (0.71 eV), leading to the experimentally recorded enhanced catalytic Faradaic efficiency of Cu-SMS. These findings underscore the crucial role of single-metal sites in Cu-SMS in promoting the NO_3_RR process, enhancing both catalytic Faradaic efficiency and selectivity. The complete reaction pathways for NO_3_RR on Cu-SMS, integrating spectroscopic and computational insights, are summarized in Fig. 6e.

## CONCLUSION

Through leveraging a novel partial-coverage-assembly strategy and graphdiyne-derived fragment ligands, we successfully synthesized and characterized atomically precise Cu-SMS molecular nanocluster catalysts. The graphdiyne-inspired bridging ligands were found to enhance intramolecular electron delocalization within each cluster while the partial surface coverage assembly minimizes inter-cluster energy barriers. Single-crystal conductivity measurements revealed that Cu-SMS exhibits over 20-fold higher conductivity than its multi-site counterpart, Cu-MMS. Cu-SMS, with its single catalytic sites, exhibited superior performance in catalyzing the reduction of NO_3_^−^ to NH_3_ compared to Cu-MMS, which contains multiple active sites. Specifically, Cu-SMS achieved an exceptional Faradaic efficiency exceeding 99% at a current density of 40 mA cm^−2^ and maintained consistent NH_3_ production over 7 h. This stability of the catalyst was confirmed through EXAFS, XPS and PXRD analysis.

The integration of *in situ* ATR-SEIRAS, ECMS and DFT calculations provided a comprehensive mechanistic understanding of NO_3_RR, revealing that single-metal sites in Cu-SMS are significantly more efficient for nitrate-to-ammonia conversion than multiple-metal sites in Cu-MMS. This enhanced efficiency stems from the precisely ordered single-metal sites, which create a uniform and well-defined catalytic microenvironment. This unique configuration improves reaction dynamics by lowering energy barriers for key reaction steps (e.g. NO* to HNO*) and enhancing selectivity toward the desired product (NH_3_). Additionally, the isolated active sites in Cu-SMS suppress side reactions, promoting near 100% Faradaic efficiency. These findings underscore the advantages of atomic-level precision and molecular ordering in designing single-site catalysts for high-performance electrochemical systems. Specifically, beyond nitrate reduction, graphdiyne-inspired bridging ligands also hold promise for heterometallic catalytic systems (e.g. CO_2_ electroreduction or oxygen reduction reaction), where they are able to promote cooperative electronic interactions between metal sites and ligand frameworks, and further boost catalytic activity and selectivity.

## Supplementary Material

nwaf575_Supplemental_Files

## Data Availability

The data supporting the findings of this study are available from the corresponding author upon reasonable request. The EXAFS data have been uploaded to the EDMOND repository and can be found at https://doi.org/10.17617/3.WYIXZT.
