# Supplementary material for: Partial-coverage assembly of graphdiyne-derived fragment-protected Cu(I) clusters generates an ordered single-metal site catalyst
Source: Natl Sci Rev. 2025 Dec 15;13(3):nwaf575. doi: 10.1093/nsr/nwaf575 (PMC12887297; doi:10.1093/nsr/nwaf575)
Supplement: nwaf575_Supplemental_Files [file nwaf575_supplemental_files.zip › NSR-Supporting_Information-20251204.pdf]

Supporting Information

Partial-Coverage-Assembly of Graphdiyne-Derived Fragment-Protected Cu(I) Clusters Creates Ordered Single-Metal Site Catalyst

Shuai Chen[1,3], Xi Fan[2*], Shuai Yan[1], Morgan McKee[1], Alexandre Terry[1], Chen Gao[1], Mahsa A bdolmaleki[1], Jost Heise[1], Minmin Chen[4], Yves Kayser[4], Serena DeBeer[4], Jian Zhang[2*], and Nikolay Kornienko[1*]

1. Institute of Inorganic Chemistry, University of Bonn, Bonn 53121, Germany

2. State Key Laboratory of Structural Chemistry, Fujian Institute of Research on the Structure of Matter, Chinese Academy of Sciences, Fuzhou 350002, P. R. China

3. Max Planck Institute for Polymer Research, Ackermannweg 10, Mainz 55128, Germany

4. Max Planck Institute for Chemical Energy Conversion, Mülheim an der Ruhr 45470, Germany

E-mail: fanxi@fjnu.edu.cn, zhj@fjirsm.ac.cn, and nkornien@uni-bonn.de

Content

Figure S1. Coordination modes of terminal ligands in σ mode (a) and σ&π modes (b)…………………………………………8

Figure S2. Powder X-ray diffraction (PXRD) measurement of Cu-SMS…………………………………………………….8

Figure S3. Powder X-ray diffraction (PXRD) measurement of Cu-MMS.…………………………………………….……8

Figure S4. The packing view of Cu-SMS along (a) a-axis and (b) c-axis. (c) The packing view of Cu-SMS along b-axis, incompletely isolated clusters result dual sites at specific boundaries, but the fraction of cluster molecules on the crystal edges is virtually negligible…………………………….………………………………………………………… ……..……9

Figure S5. (a) Single crystal conductivity instrument. (b) Single crystal during the measurement. ……………………..…9

Figure S6. ^{1}H NMR spectrum of NH_3 product at –0.8 V (a), –1.0 V (b), –1.2 V (c), –1.4 V (d), and –1.6 V (a) vs. RHE in Cu-SMS.. …………………………………………………………………………….……………………………10

Figure S7. ^{1}H NMR spectrum of NH_3 product at –0.8 V (a), –1.0 V (b), –1.2 V (c), –1.4 V (d), and –1.6 V (a) vs. RHE in Cu-MMS.. ………...10

Figure S8. The UV-vis absorption spectra for NO_2^- product at –0.8 V (a), –1.0 V (b), –1.2 V (c), –1.4 V (d), and –1.6 V (a) vs. RHE in Cu-SMS.. ………………………………………………………………………………………..…11

Figure S9. The UV-vis absorption spectra for NO_2^- product at –0.8 V (a), –1.0 V (b), –1.2 V (c), –1.4 V (d), and –1.6 V (a) vs. RHE in Cu-SMS.. ………………………………………………………………………………………..…11

Figure S10. FE of ammonia and nitrite products at different applied potentials in Cu-SMS (a) and Cu-MMS (b)…………..…11

Figure S11. a The UV-vis absorption spectra for standard KNO_2 solutions with different concentrations. b The corresponding calibration curve for the colorimetric NO_2^- assay..…………………………………………………………………………12

Figure S12. The calibration curve of NH_3 products. The corresponding concentrations are linearly correlated with the integral area of their characteristic peaks. .... ……………………………………………………………….……….12

Figure S13. Controlled experiments for Cu-SMS catalysts………………………………………………………….…….12

Figure S14. The survey XPS spectrum and the image from Nexsa load lock camera of Cu-SMS with −0.8 V vs RHE………14

Figure S15. The survey XPS spectrum and the image from Nexsa load lock camera of Cu-SMS with −1.0 V vs RHE………. **错误!未定义书签。**

Figure S16. The survey XPS spectrum and the image from Nexsa load lock camera of Cu-SMS with −1.2 V vs RHE……… 错误!未定义书签。

Figure S17. The survey XPS spectrum and the image from Nexsa load lock camera of Cu-SMS with −1.4 V vs RHE……….15

Figure S18. Cu LMM Auger spectra were obtained at –0.8 V (a), –1.0 V (b), –1.2 V (c), and –1.4 V (d) vs. RHE in Cu-MMS…….. …17

Figure S19. Quantitative analysis of Cu LMM Auger spectra peak positions recorded at –0.8 V, –1.0 V, –1.2 V, and –1.4 V vs. RHE for Cu-MMS, compared with reference oxidation states (Cu^0, Cu^+, Cu^{2+}) from Cu, Cu_2O, CuO standards………...17

Figure S20. Cu k-space EXAFS extracted from the acquired data of Cu-SMS with different working conditions……………17

Figure S21. XRD pattern of Cu-SMS catalyst before and after NO_3RR.. ………………………………………… ……… 错误!未定义书签。

Figure S22. The TEM images of Cu-SMS and Cu-MMS before and after the electrochemical NO_3RR. (a) Cu-SMS before test, (b) Cu-SMS after test, (c) Cu-MMS before test, (d) Cu-MMS after test...…………25

Figure S23. The SEM images (a), mapping (b-f), EDS (g, e) of Cu-SMS before grinding sample preparation...…………25

Figure S24. The SEM images (a), mapping (b-f), EDS (g, e) of Cu-MMS before grinding sample preparation...…………25

Figure S25. The SEM images (a), mapping (b-f), EDS (g, e) of Cu-SMS on carbon paper before test...…………20

Figure S26. The SEM images (a), mapping (b-f), EDS (g, e) of Cu-SMS on carbon paper after test...…………21

Figure S27. The SEM images (a), mapping (b-f), EDS (g, e) of Cu-MMS on carbon paper before test...…………22

Figure S28. The SEM images (a), mapping (b-f), EDS (g, e) of Cu-MMS on carbon paper after test...…………23

Figure S29. Potential-dependent *in-situ* ATR-SEIRAS on the Cu-SMS surfaces using $K^{14}NO_3$ solutions..…………………24

Figure S30. Potential-dependent in-situ ATR-SEIRAS on the Cu-SMS surfaces using $K^{15}NO_3$ solutions..…………………24

Figure S31. The Gibbs free energy diagrams for NO_3RR on Cu-SMS along the optimal pathway..…………………………25

Figure S32. The Gibbs free energy diagrams for NO_3RR on Cu-MMS along the optimal pathway……………………….. 25

Table S1. Crystal data for Cu-SMS……………………………………………………………………………..…26

Table S2. Possible band assignments of *in-situ* ATR-SEIRAS………………………………………………………….……27

Table S3. The element content of the sample from −0.8 V to −1.4 V vs RHE based on the XPS spectrum…………………28

Table S4. The comparison of the NH3 synthesis activity of Cu-SMS in NO_3RR with other catalysts under ambient conditions..………………………………………………………………………………………………………..…28

References……..30

Experimental Section

Materials and Measurements.

All reagents and solvents used in this work were commercially sourced and used without further purification. Toluene, trifluoroacetic acid, ethanol, and acetone were obtained from Sigma Aldrich. Cu_2O and phenylacetylene were supplied by TCI Chemicals. 1,4-Diphenylbutadiyne was supplied by Adamas-beta®. $KHCO_3$ (extra pure) and N, N-dimethylformamide (> 99%) were supplied by Fisher Scientific. KNO_3 (99%) was purchased from AppliChem GmbH. $Cu(NO_3)_2.3H_2O$ and 1H-pyrazole-4-carbaldehyde were provided by Abcr GmbH, while Nafion was supplied by Ion Power GmbH. The resistance of deionized water was 18.2 MΩ cm^{-1}. Agilent Cary 60 UV-Vis spectrophotometer was used for UV-Vis absorption measurements. ^{1}H NMR was recorded on a Bruker 500 MHz NMR instrument. The conductivity of single crystal was measured on the Lakeshore CRX-VF Cryogenic Probe Station. XPS was conducted on a Thermo Scientific Nexsa G2. In-situ FTIR spectra were collected using a VERTEX 80 spectrometer from Bruker. *In-situ* DEMS data was collected using with integrated mass spectrometer from Spectro Inlet.

Molecular Cu Catalyst Synthesis

Preparation of Cu-SMS molecular catalyst: Cu_2O (0.1g, 0.7 mmol) was added to toluene (8 mL), followed by the dropwise addition of trifluoroacetic acid (0.1 mL, 1.3 mmol) and 1,4-diphenylbutadiyne (0.1 mL, 0.9 mmol). The resulting mixture was heated at 85 °C for 24 hours. After cooling to room temperature for over 24 hours, the mixture was washed with toluene, and red crystals of Cu-SMS were obtained. (yield: 50% based on Cu_2O)

Preparation of Cu-MMS molecular catalyst: $Cu(NO_3)_2.3H_2O$ (0.20g, 0.83 mmol), 1H-pyrazole-4-carbaldehyde (0.096 g, 1.0 mmol), N, N-dimethylformamide (6.7 mL), deionized water (5.0 mL), and ethanol (6.7 mL) were added to a 25 mL vial. The mixture was heated at 100 °C for 12 hours, resulting in the formation of light yellow single crystals. The crystals were then washed three times with acetone. (yield: 70% based on $Cu(NO_3)_2.3H_2O$)

Single-Crystal X-ray Diffraction (SCXRD) Studies

The structure determination of Cu-SMS was collected on a Supernova single crystal diffractometer equipped with graphite-monochromatic Cu K radiation (λ =1.54178 Å) at 150 K or room temperature. Absorption correction was applied using SADABS [1]. Using Olex2 [2], the structure was solved with the SHELXT [3], structure solution program using Intrinsic Phasing and refined with the SHELXL, refinement package using Least Squares minimization. Crystal data and details of data collection and refinement of Cu-SMS are in Table S1. 2403302 (Cu-SMS) contains the supplementary crystallographic data for this paper. This data is provided free of charge by The Cambridge Crystallographic Data Centre.

Powder X-ray Diffraction (PXRD)

PXRD was obtained on a STOE STADI P powder diffraction instrument equipped with a MYTHEN2 R 1K silicon strip detector from a 2θ range of 5–90° using a CoKa1 (1.788965 Å) source. Furthermore, the CoKa1 2θ values are converted to CuKa1 (1.540562 Å).

Transmission Electron Microscopy (TEM)

TEM samples were prepared by drop-casting the ethonal dispersion onto a carbon-coated copper grid. Routine TEM imaging was done using JEOL1400 TEM with an acceleration voltage of 120 kV.

Scanning Electron Microscopy (SEM)

SEM was using the Hitachi SU3800 instrument equipped with an Octane Elect EDS system for enhanced energy-dispersive X-ray spectroscopy (EDAX) mapping.

Extended X-ray Absorption Fine Structure (EXAFS) Spectroscopy

The EXAFS data at the Cu K-edge were acquired in transmission mode using a laboratory-based X-ray spectrometer in a von Hamos geometry with a tilted detector arrangement. The basic geometry of the spectrometer corresponds to that described in Ref.[4], the main difference being that the crystal and detector are installed in a vacuum housing and operated at a pressure of about 0.01 mbar. The instrument is optimized for EXAFS experiments in the sense that the resolving power has been compromised for higher detection efficiency and bandwidth, i.e., the resolving power $E/\Delta E$ is estimated to be around 1800 for measurements at the Cu K-edge. A 40 µm thick, highly annealed pyrolytic graphite crystal [5] mounted on a cylindrically curved glass substrate with a bend radius of 100 mm is used as dispersive optics and an EIGER hybrid photon pixel counting detector as position position-sensitive detector [6]. In combination with a polychromatic and divergent X-ray source, these two components allow by their dispersive-type arrangement for a scan-free measurement, where the EXAFS data are collected by integration over time. For the current experiment, the monitored bandwidth was around 3 keV and centered on the Cu K-edge. The spectrometer source was a micro-focused X-ray tube with a Mo anode that was operated at 17.3 keV and 1.8 mA. This high voltage setting suppressed X-ray photons that would fulfill the second-order diffraction condition for Bragg angles in the Cu K EXAFS range in the first diffraction order. The X-ray source size was approximately 70 µm.

For the EXAFS measurements, the Cu-SMS molecular catalysts were prepared under similar conditions to those used in the electrochemical reactions, ranging from the fresh state to −1.6 V vs. RHE. The XAS samples were then prepared as 13 mm diameter pellets after homogenization of 20 mg of the molecular catalyst powders with 60 mg of a binder (N,N'-ethylenebis (stearamide), $C_{38}H_{76}N_2O_2$). The pellets were positioned approximately 1 cm from the Be-window of the X-ray tube on the path of the Bremsstrahlung spectrum towards the crystal. During the measurement, the samples were continuously moved back and forth by 1 mm on an axis perpendicular to the X-ray propagation direction and within the dispersion plane of the spectrometer in order to mitigate any residual inhomogeneities. In this way, each energy bin of the processed spectra is averaged over a larger sample volume. Data were collected between 77 and 87 minutes for I_0 (reference spectrum without sample) and between 106 and 121 minutes for I_t (spectrum with sample in the beam path). For the Cu foil of 6 µm thickness the corresponding approximate measurement times were 7 minutes and 14 minutes. The integrated 2D images were processed to assign a corresponding X-ray photon energy to each detector pixel based on geometric considerations from spectrometer alignment and image evaluation. The necessary processing steps are described in Ref.[7] and an energy bin of 2 eV was chosen for the presented data.

The final spectra were calibrated by aligning the E_0 of a reference Cu foil to 8980.3 eV. The XAS data were processed using the Athena module within the Demeter software package [8]. A first-order polynomial and a second-order polynomial were applied to the pre-edge and post-edge regions, separately, to subtract the background. The k-range 2-12 $Å^{-1}$ was used for Fourier transforms in EXAFS. The resultant EXAFS was k^2-weighted to enhance the impact of high-k data.

Wavelet transform analysis of the real EXAFS spectrum was performed using the HAMA software. The Morlet wavelet function was selected as the mother wavelet. The parameters used for the wavelet transform were: KappaMorlet = 10 and SigmaMorlet = 1. These values were chosen to balance spatial resolution in both k and R domains.

Electrochemical Reactions

To prepare the working electrode, 10 mg of the molecular catalyst was mixed with 620 μL of ethanol, 300 μL of deionized water, and 80 μL of Nafion. After sonication for 10 min, the resulting catalyst ink was evenly applied to carbon paper.

Electrochemical NO_3RR measurements were carried out using an electrochemical workstation (SP200, EC-lab) in a three-electrode configuration. The setup consisted of catalyst-coated carbon paper as the working electrode, an Ag/AgCl reference electrode, and a graphite rod as the counter electrode. The reactions were performed at room temperature in a 0.5 M $KHCO_3$ and 0.2 M KNO_3 solution within a three-neck glass bottle. All electrode potentials were converted to the reversible hydrogen electrode (RHE) reference scale using the following equation:

$$E\ (vs.\ RHE) = E\ (vs.\ Ag/AgCl) + 0.059 \times pH + 0.197\ V$$

Product Qualification

NH_3 was quantified using ^{1}H-NMR spectroscopy with a water suppression mode. A standard maleic acid solution in DMSO was used to determine the product concentrations. The Faradaic efficiency (FE) and partial current density (j) were calculated using the following equations:

$$FE = cVzF/Q\times100\%;\ j = Q/t_{total}\times FE$$

In those equations, z represents the number of electrons transferred, c is the concentration of the liquid products, V is the volume of the electrolyte. F is the Faraday constant, Q is the total charges passed, and t_{total} is the total duration of the experiment.

The nitrite concentration was determined using the standard Griess method. In this process, the Griess reagent was mixed with the electrolyte, and the mixture was allowed to react for 10 min. The absorbance of the solution was then measured at 519 nm using UV-Vis spectroscopy.

Electrochemical infrared spectroscopy

Fourier transform infrared (FT-IR) spectra were collected on a Bruker VERTEX 80 spectrometer over the range 400−4000 cm^{-1}. In-situ ATR-SEIRAS measurements were performed using a Pike Veemax setup equipped with a Si ATR crystal coated with a diamond surface. The infrared pulse was incident at an angle of 55°. Absorbance spectra were calculated using the equation $A = -\log(I_1/I_2)$, where I_1 and I_2 represent the intensities of the incident and reflected pulse, respectively. Typically, 15

scans were recorded for each measurement. The experiment utilized a three-electrode cell configuration, with an Ag/AgCl reference electrode, a graphite rod as the counter electrode, and a Si ATR crystal coated with an Au film and molecular Cu catalysts serving as the working electrode. The electrolyte consisted of 0.5M $KHCO_3$ and 0.2M KNO_3, saturated with Ar gas.

Electrochemical Mass Spectrometry (ECMS)

In-situ ECMS (Spectro Inlet) testing was performed using an electrochemical cell containing $KHCO_3$ and KNO_3 electrolytes operated with an SP150 electrochemical workstation (EC-lab). The setup employed a carbon-coated molecular Cu catalyst as the working electrode, a graphite rod as the counter electrode, and an Ag/AgCl electrode as the reference. Mass spectrometry signals were monitored concurrently with cyclic voltammetry, which was conducted at a scan rate of 5 mV s^{-1}.

Computational Details

DFT calculations were carried out using the Vienna ab initio Simulation Program (VASP) [9,10]. The generalized gradient approximation (GGA) with the Perdew-Burke-Ernzerhof (PBE) functional was employed, along with a plane-wave basis set with a cutoff energy of 500 eV [11]. A 2 × 2 × 2 Monkhorst-Pack grid was used for Brillouin zone sampling during structure optimization [12]. Ion-electron interactions were modeled using the projector augmented wave (PAW) method [13]. Convergence criteria for structural optimization were set to ensure that the maximum force on any atom was less than 0.02 eV/Å and the total energy change was below 1×10^{-5} eV. Dispersion interactions were accounted for using the DFT-D3 semiempirical correction based on Grimme's scheme [14]. Gibbs free energy change (ΔG) for individual reaction steps was calculated using the following equation:[15,16]

$$\Delta G = \Delta E + \Delta ZPE - T\Delta S + \Delta G_U + \Delta G_{\mathrm{pH}}$$

Here, ΔE and ΔZPE represent the adsorption energy from density functional theory calculations and the zero-point energy correction, respectively. T, ΔS, U, and ΔG_{pH} denote the temperature, entropy change, applied electrode potential, and free energy correction of the pH, respectively.

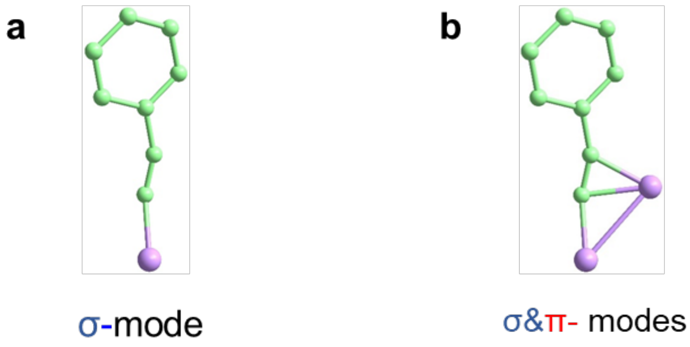

Figure S1. Coordination modes of terminal ligands in σ mode (a) and σ&π modes (b).

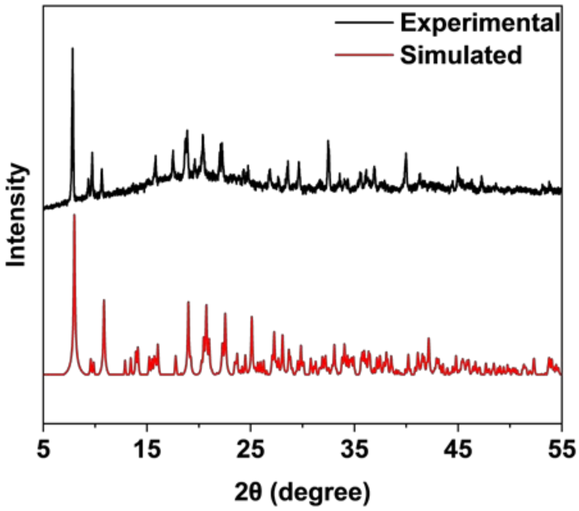

Figure S2. Powder X-ray diffraction (PXRD) measurement of Cu-SMS.

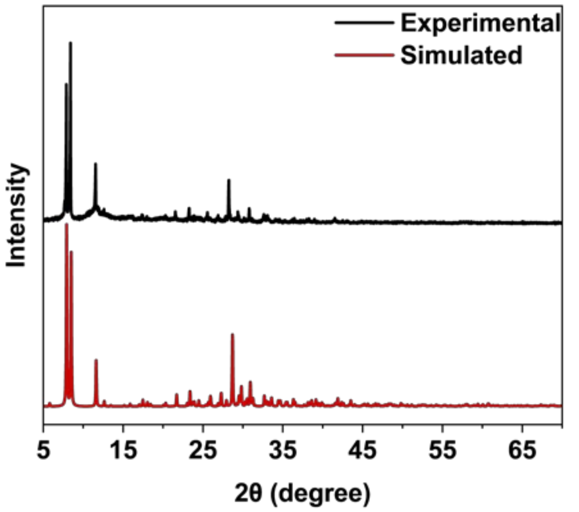

Figure S3. Powder X-ray diffraction (PXRD) measurement of Cu-MMS.

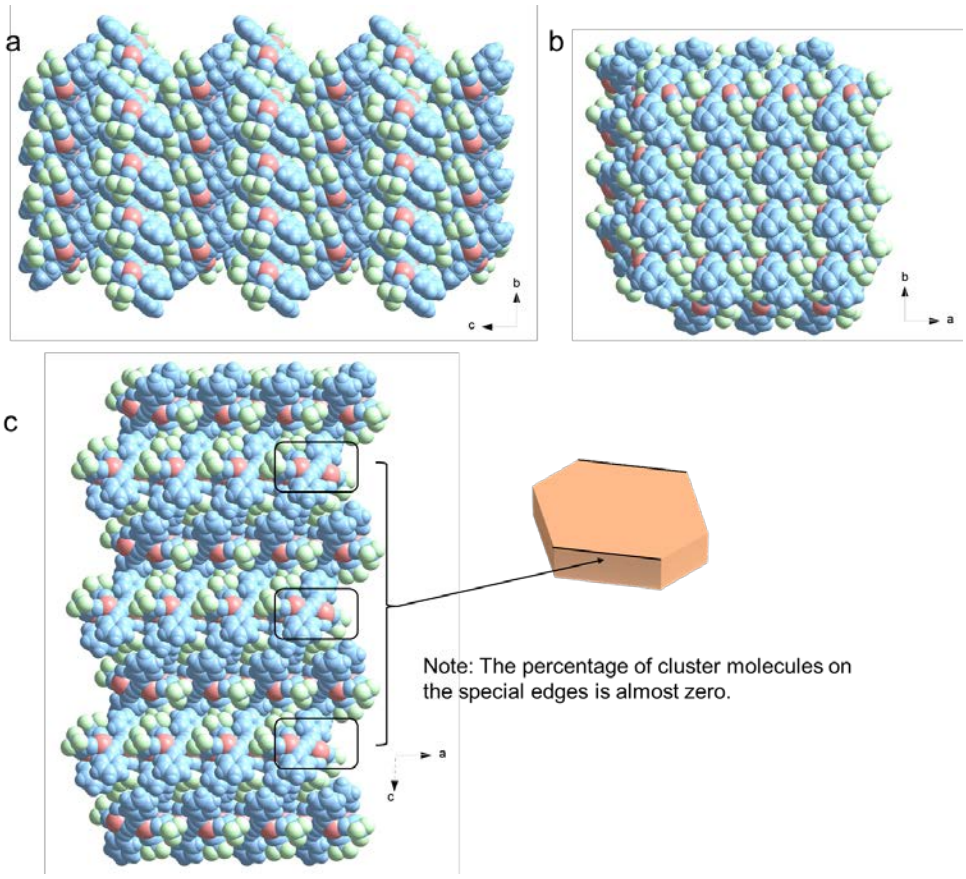

Figure S4. The packing view of Cu-SMS along (a) a-axis and (b) c-axis. (c) The packing view of Cu-SMS along b-axis, incompletely isolated clusters result dual sites at specific boundaries, but the fraction of cluster molecules on the crystal edges is virtually negligible.

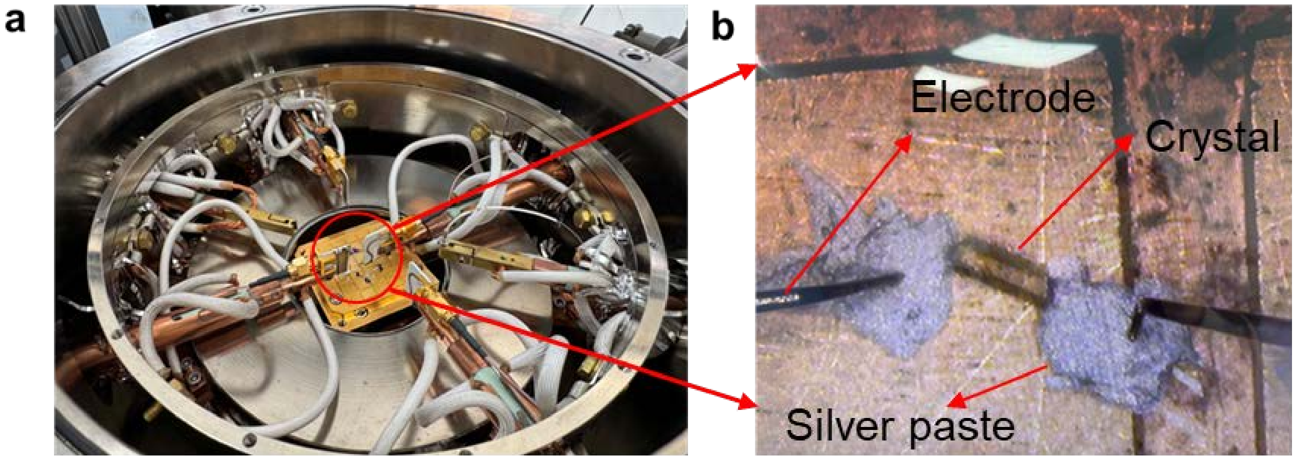

Figure S5. (a) Single crystal conductivity instrument. (b) Single crystal during the measurement.

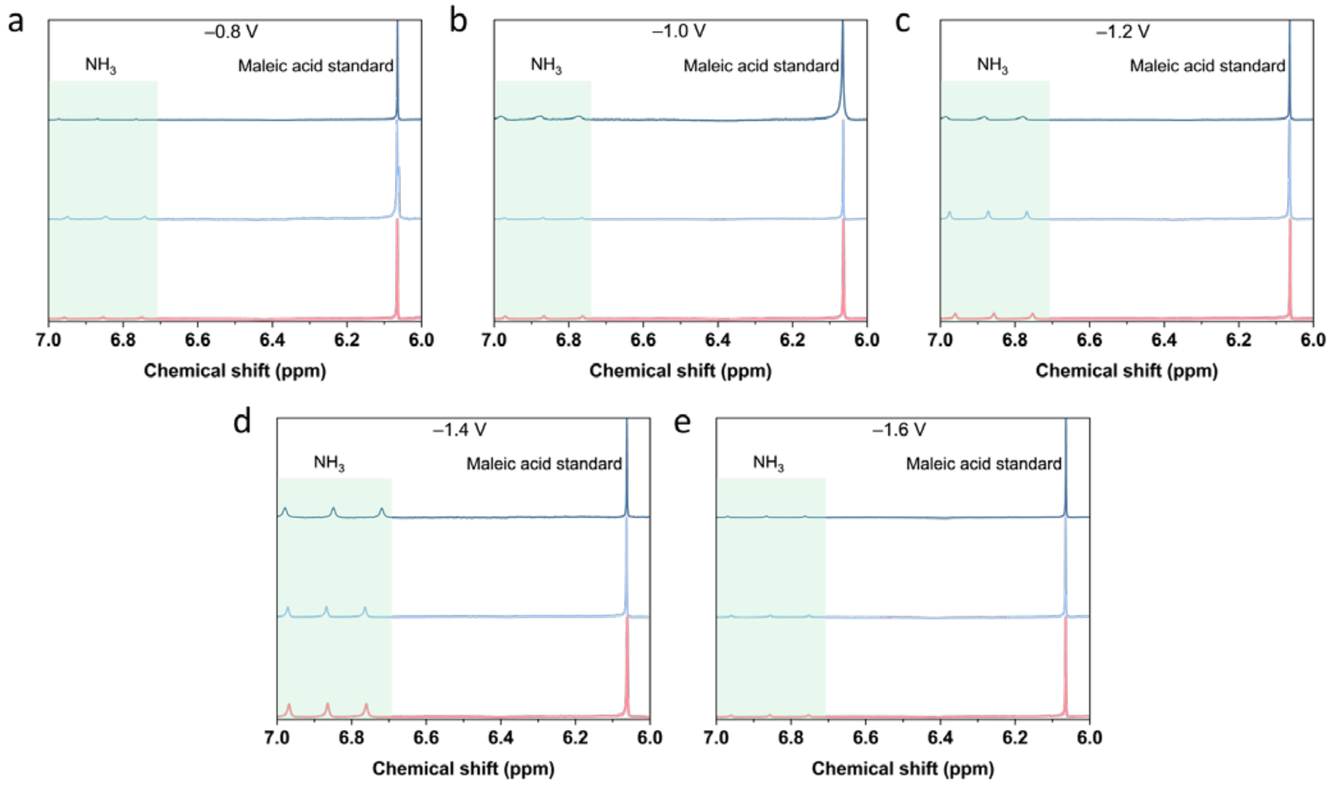

Figure S6. ^{1}H NMR spectrum of NH_3 product at –0.8 V (a), –1.0 V (b), –1.2 V (c), –1.4 V (d), and –1.6 V (a) vs. RHE in Cu-SMS.

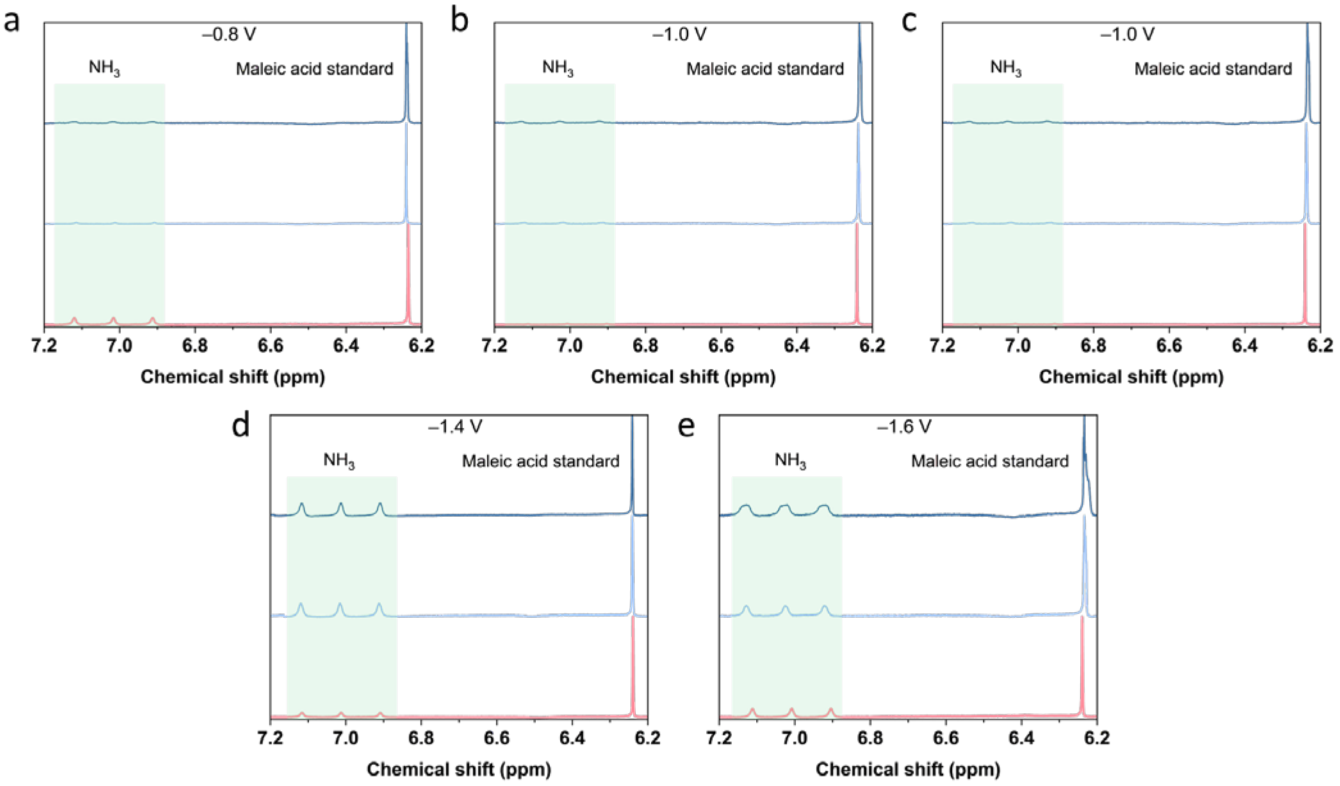

Figure S7. ^{1}H NMR spectrum of NH_3 product at –0.8 V (a), –1.0 V (b), –1.2 V (c), –1.4 V (d), and –1.6 V (a) vs. RHE in Cu-MMS.

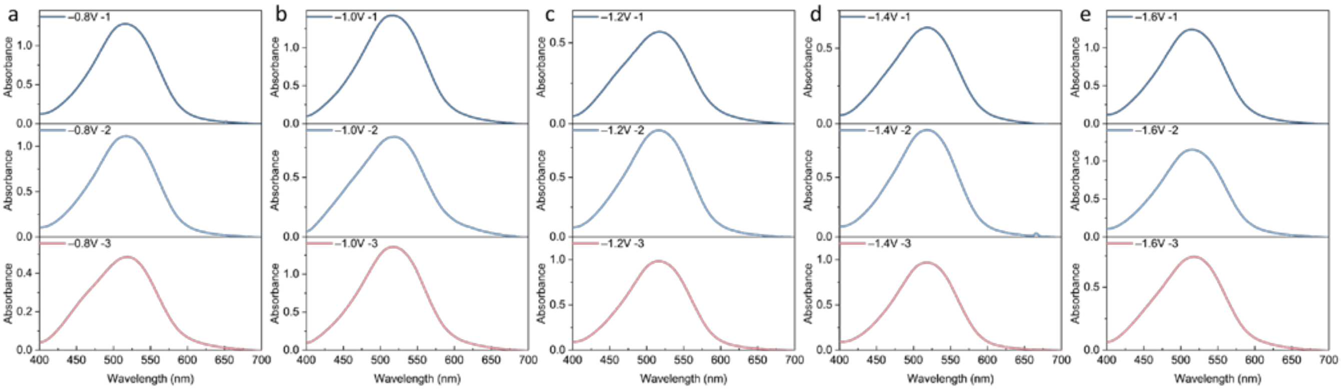

Figure S8. The UV-vis absorption spectra for NO_2^- product at –0.8 V (a), –1.0 V (b), –1.2 V (c), –1.4 V (d), and –1.6 V (a) vs. RHE in Cu-SMS.

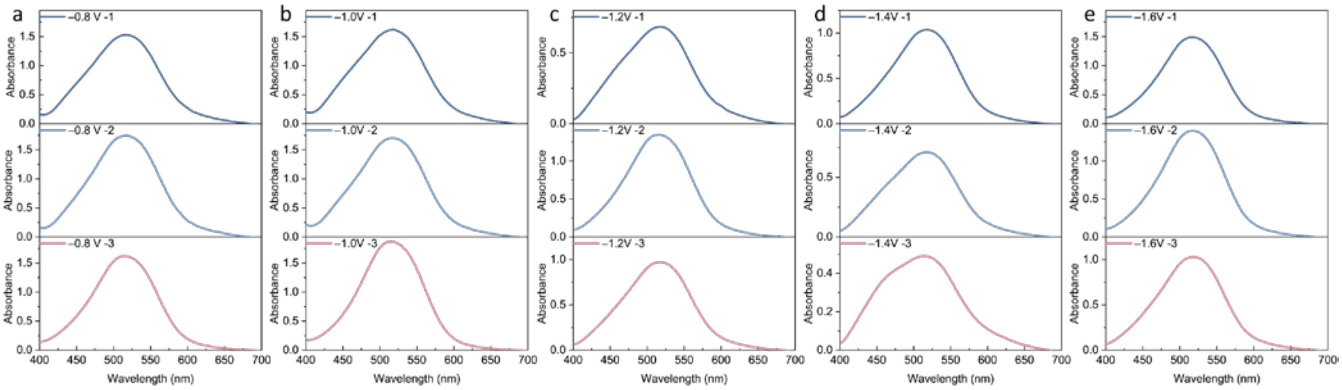

Figure S9. The UV-vis absorption spectra for NO_2^- product at –0.8 V (a), –1.0 V (b), –1.2 V (c), –1.4 V (d), and –1.6 V (a) vs. RHE in Cu-MMS.

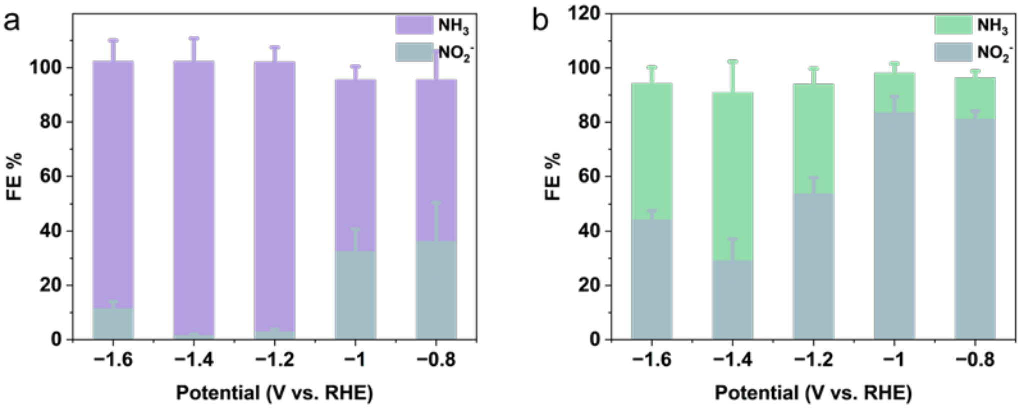

Figure S10. FE of ammonia and nitrite products at different applied potentials in Cu-SMS (a) and

Cu-MMS (b).

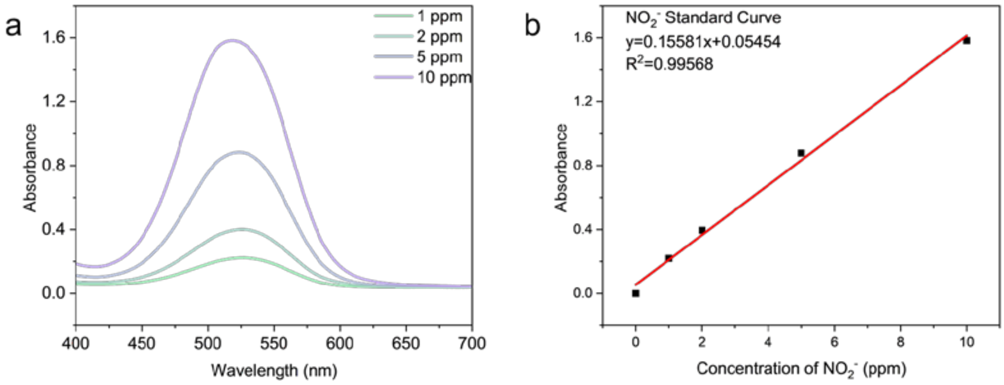

Figure S11. (a) The UV-vis absorption spectra for standard KNO_2 solutions with different concentrations. (b) The corresponding calibration curve for the colorimetric NO_2^- assay.

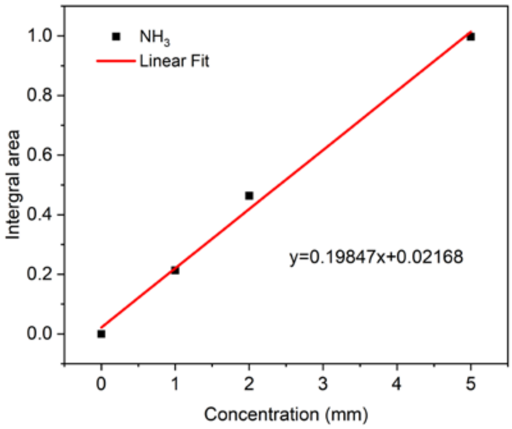

Figure S12. The calibration curve of NH_3 products. The corresponding concentrations are linearly correlated with the integral area of their characteristic peaks.

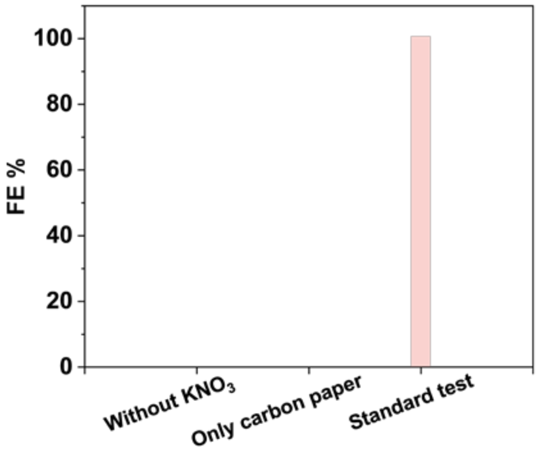

Figure S13. Control experiments for Cu-SMS catalysts.

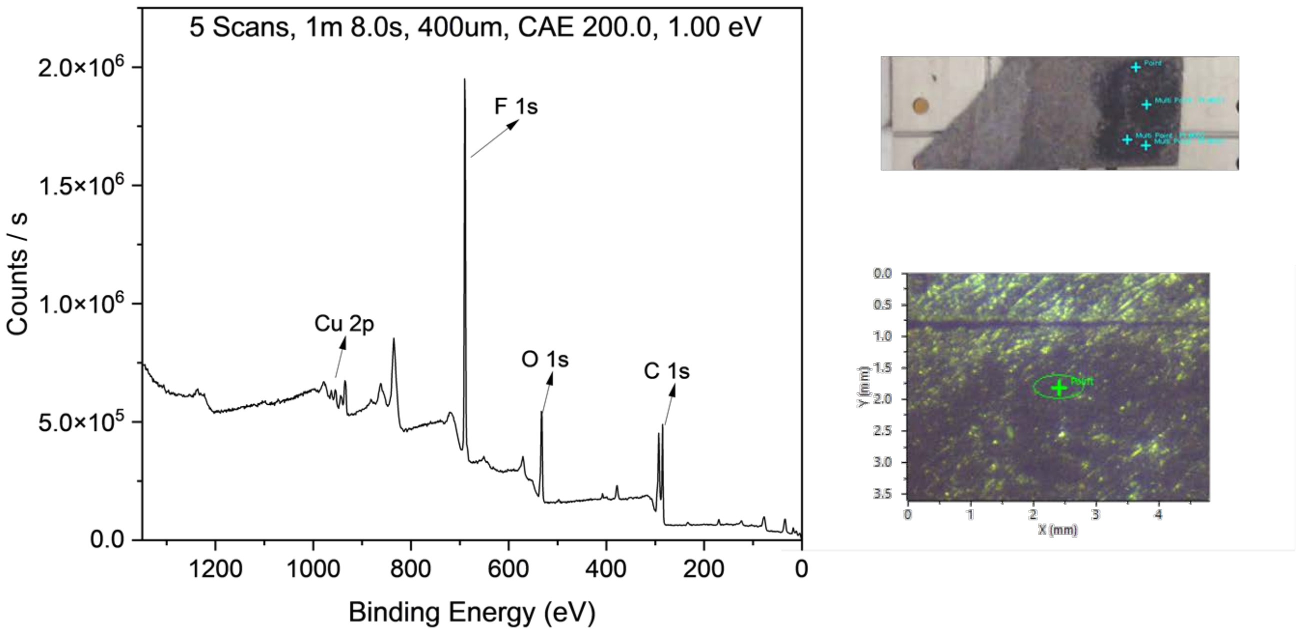

Figure S14. The survey XPS spectrum and the image from Nexsa load lock camera of Cu-SMS with −0.8 V vs RHE.

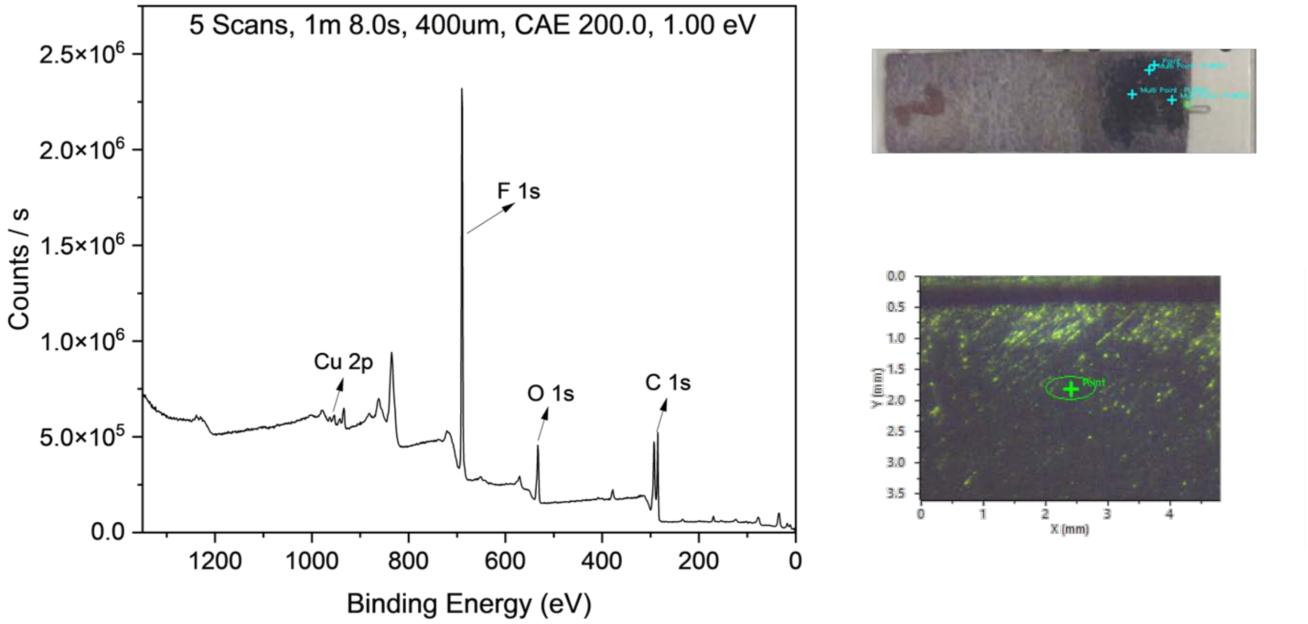

Figure S15. The survey XPS spectrum and the image from Nexsa load lock camera of Cu-SMS with −1.0 V vs RHE.

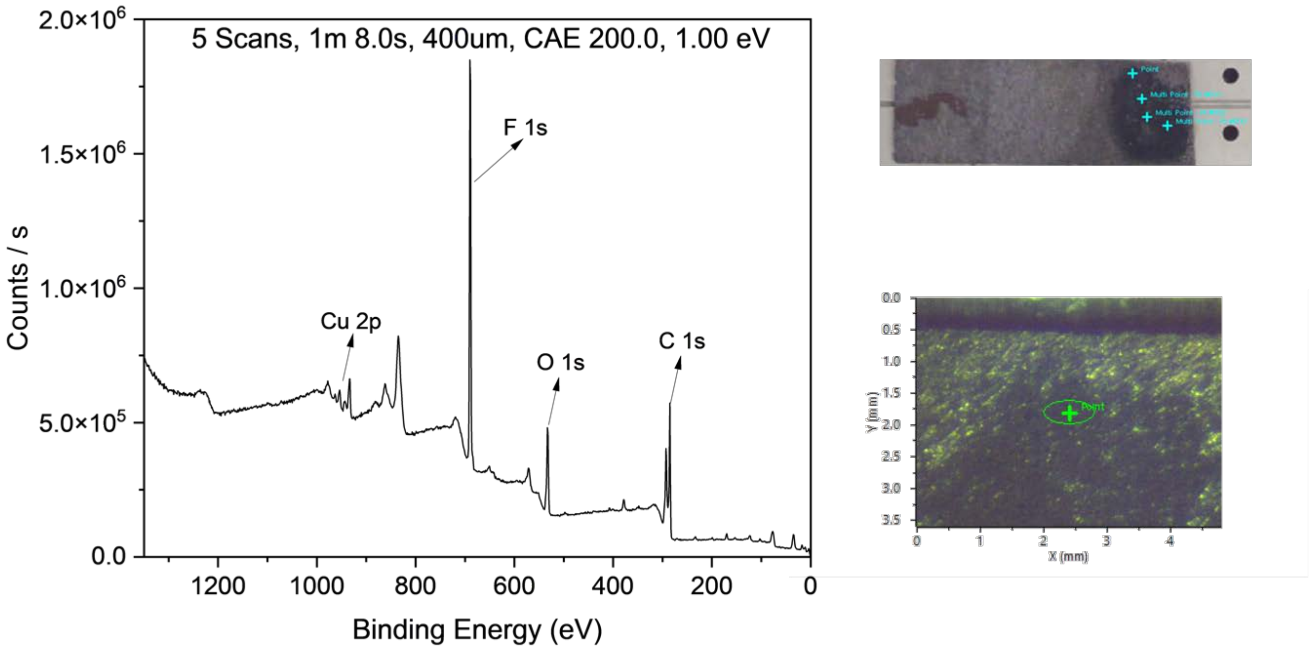

Figure S16. The survey XPS spectrum and the image from Nexsa load lock camera of Cu-SMS with −1.2 V vs RHE.

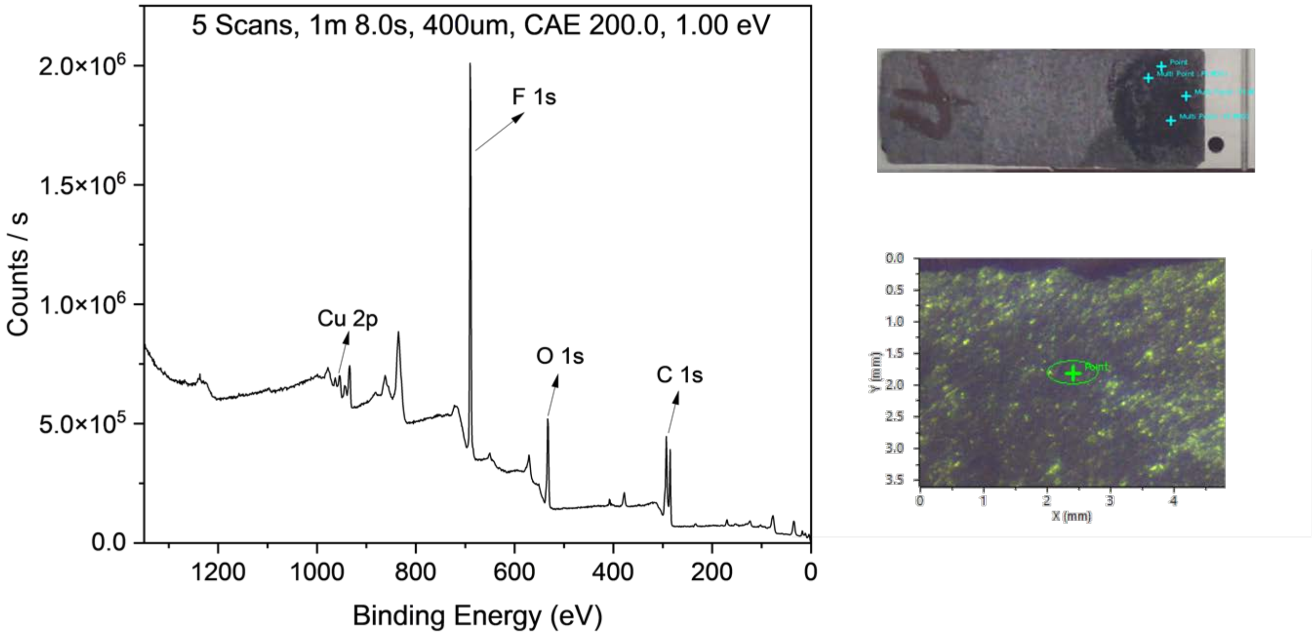

Figure S17. The survey XPS spectrum and the image from Nexsa load lock camera of Cu-SMS with

−1.4 V vs RHE.

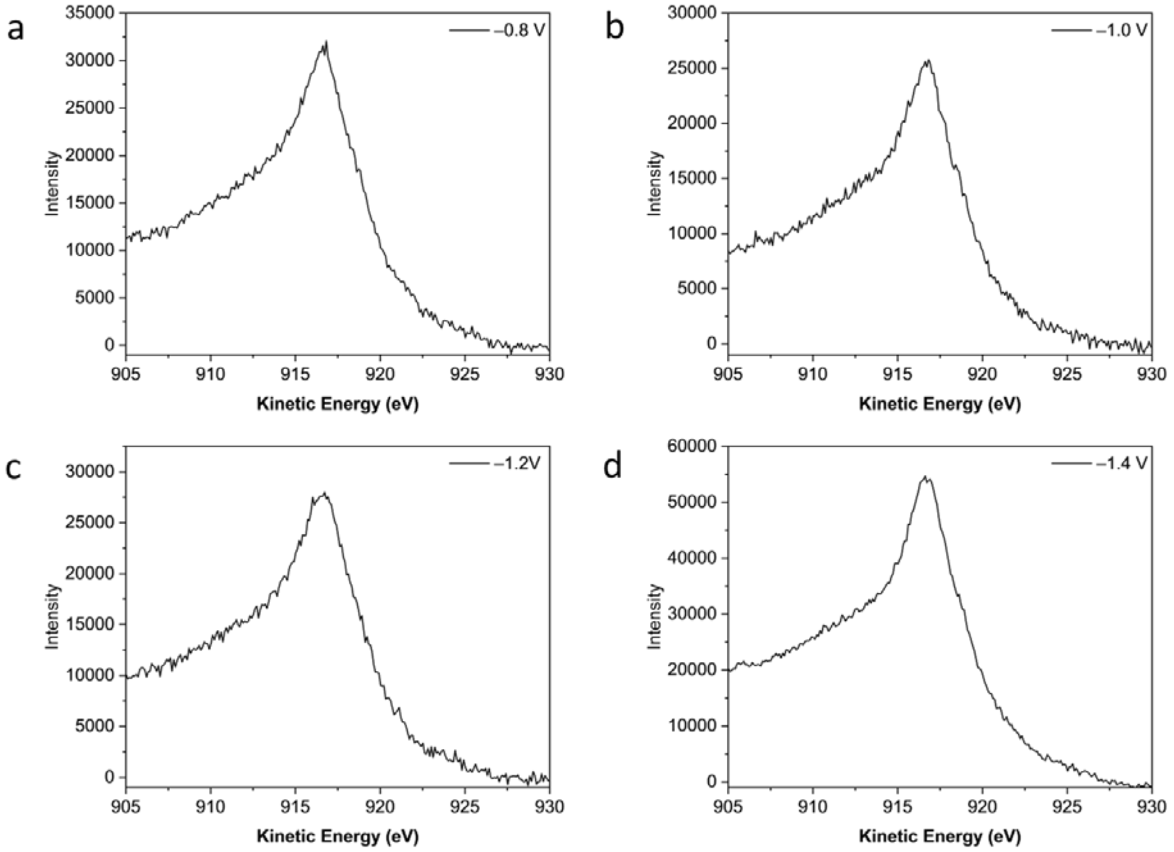

Figure S18. Cu LMM Auger spectra were obtained at –0.8 V (a), –1.0 V (b), –1.2 V (c), and –1.4 V (d) vs. RHE in Cu-MMS.

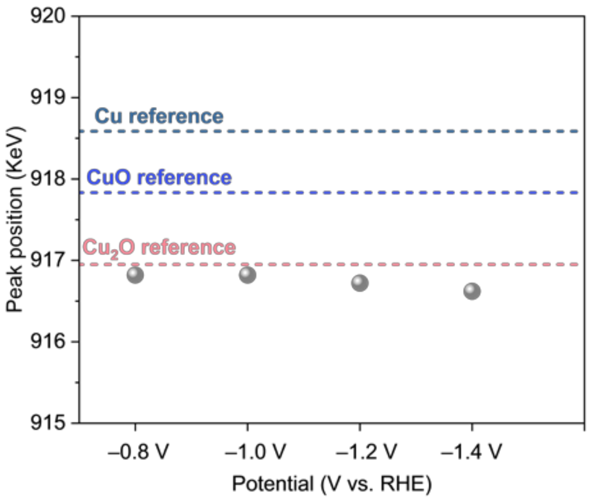

Figure S19. Quantitative analysis of Cu LMM Auger spectra peak positions recorded at –0.8 V, –1.0 V, –1.2 V, and –1.4 V vs. RHE for Cu-MMS, compared with reference oxidation states (Cu^0, Cu^+, Cu^{2+})

from Cu, Cu_2O, CuO standards.

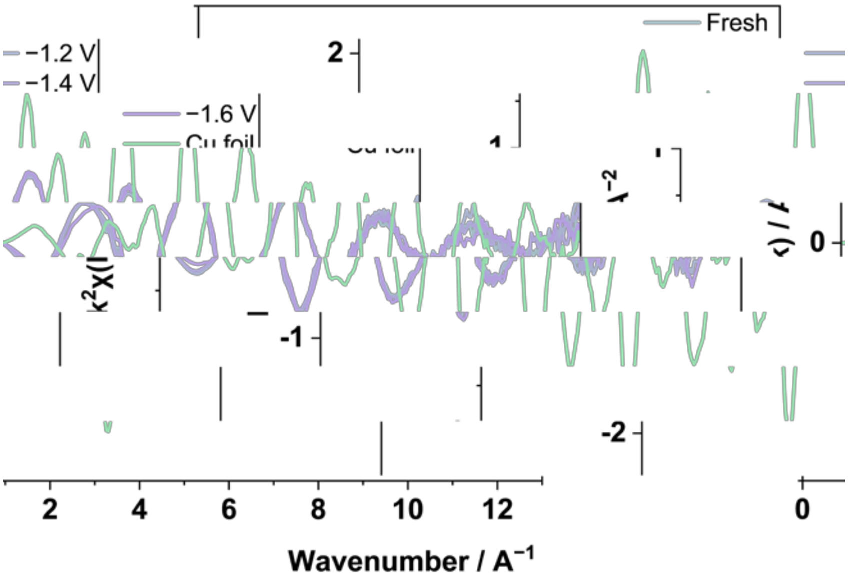

Figure S20. Cu k-space EXAFS extracted from the acquired data of Cu-SMS with different working conditions.

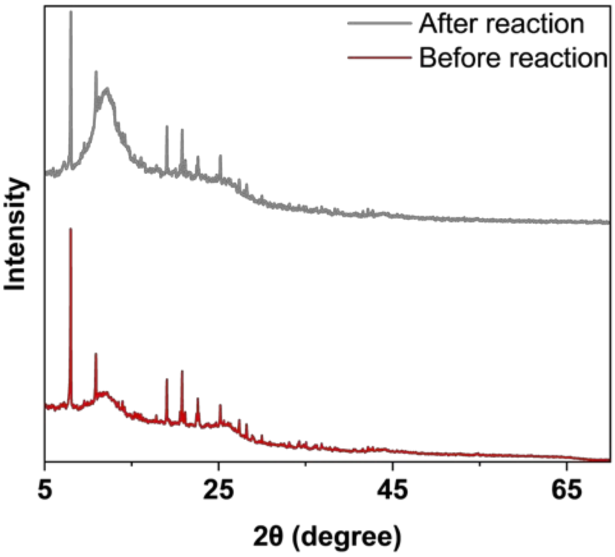

Figure S21. XRD pattern of Cu-SMS catalyst before and after NO_3RR.

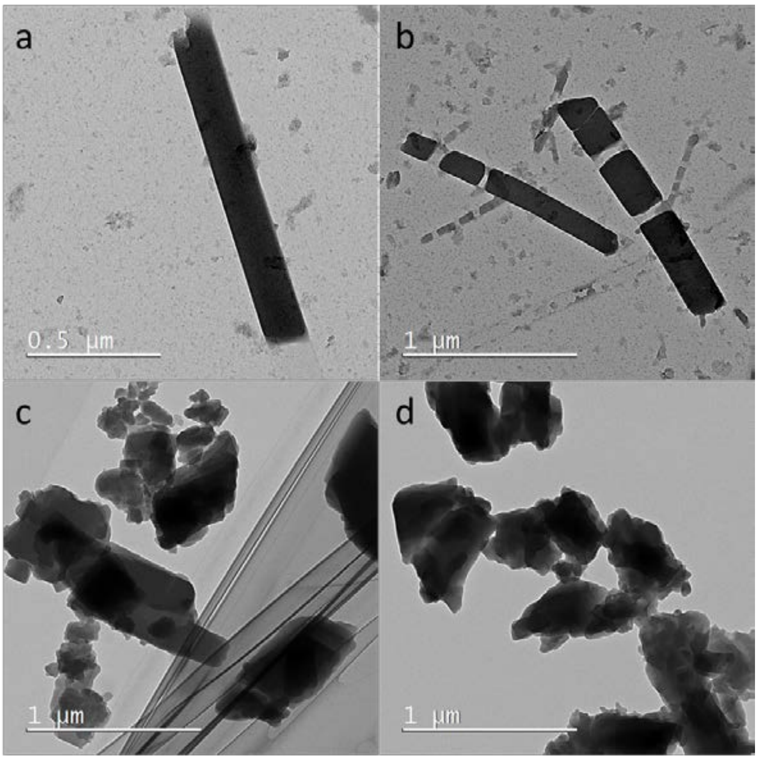

Figure S22. The TEM images of Cu-SMS and Cu-MMS before and after the electrochemical NO_3RR. (a) Cu-SMS before test, (b) Cu-SMS after test, (c) Cu-MMS before test, (d) Cu-MMS after test.

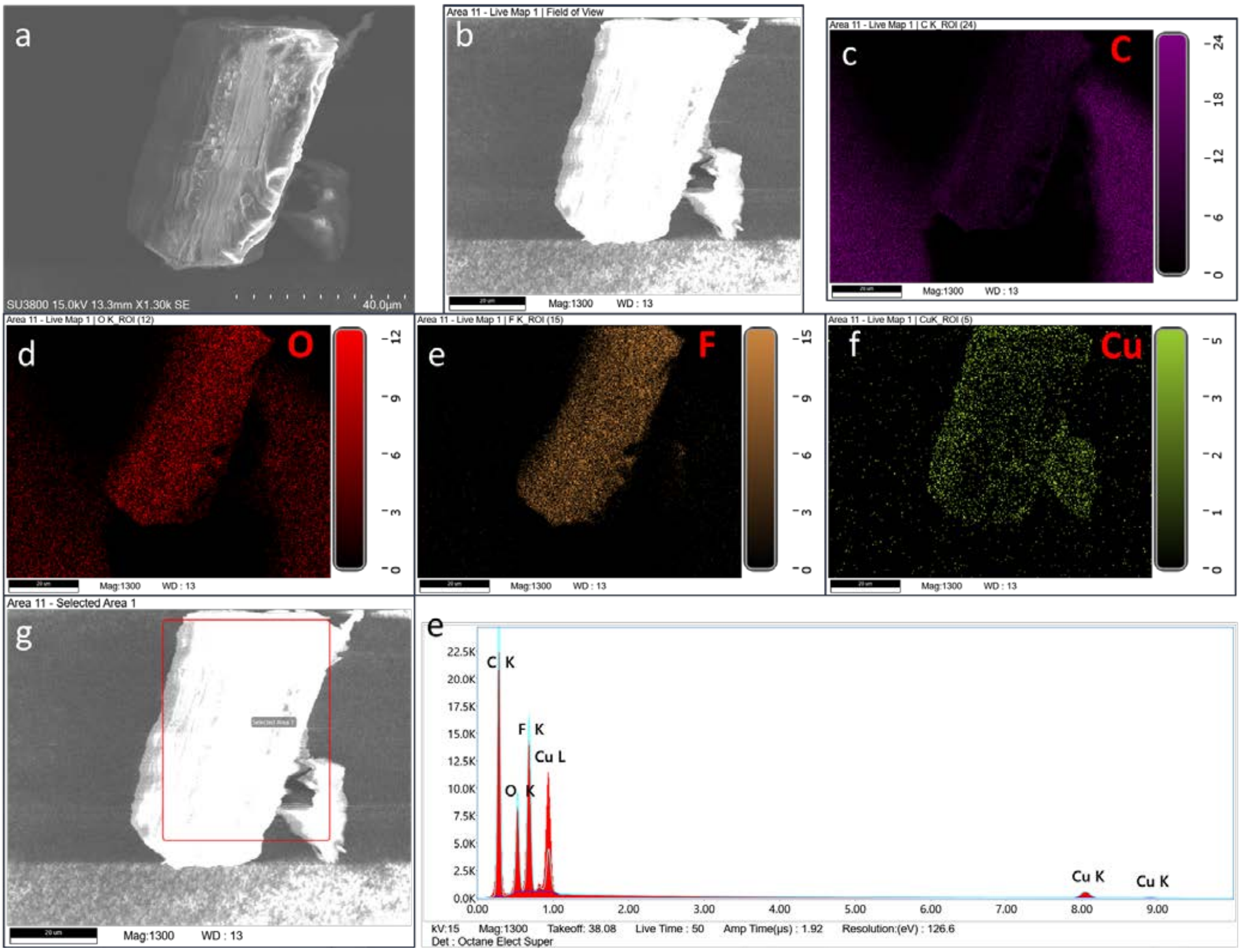

Figure S23. The SEM images (a), mapping (b-f), EDS (g, e) of Cu-SMS before grinding sample preparation.

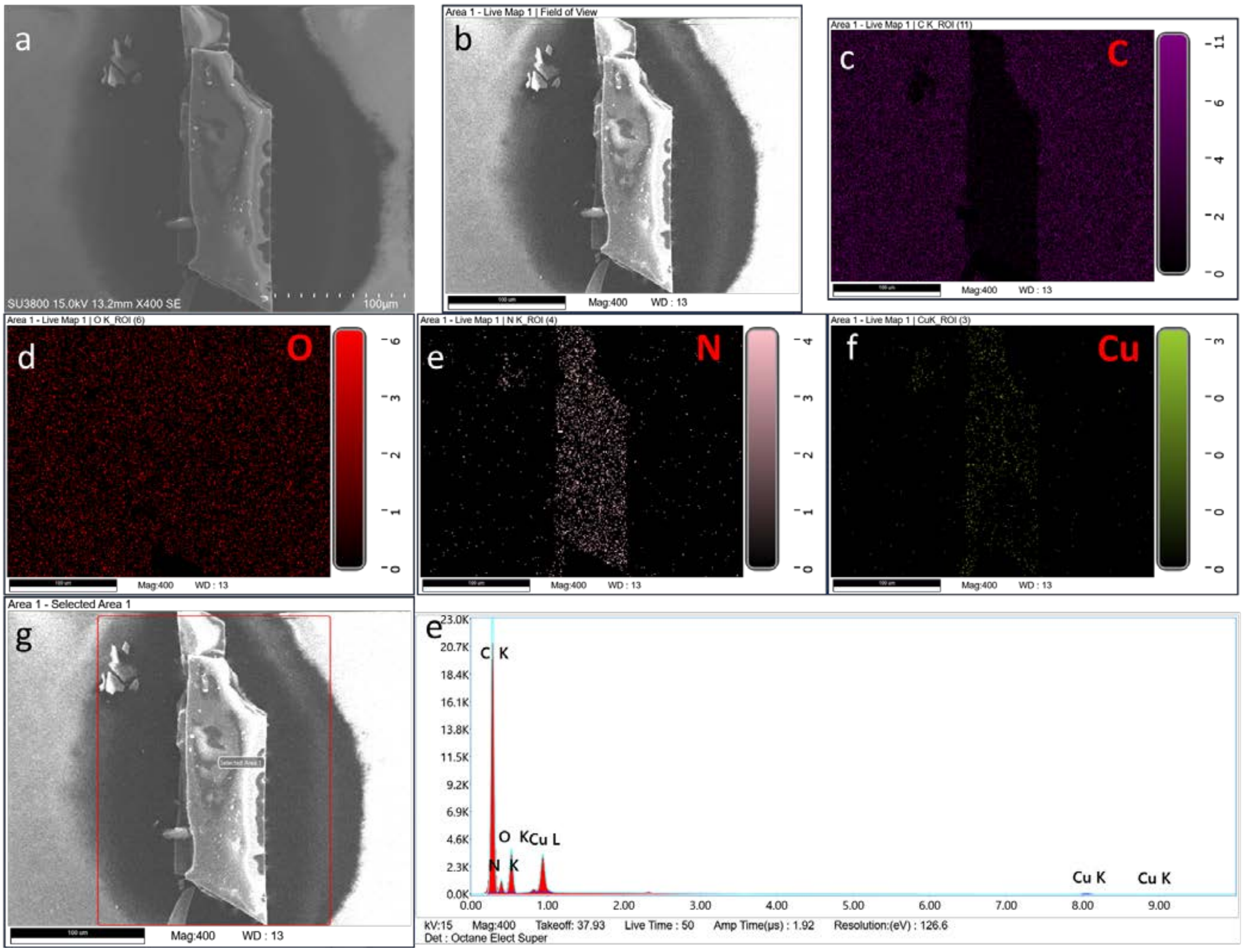

Figure S24. The SEM images (a), mapping (b-f), EDS (g, e) of Cu-MMS before grinding sample preparation.

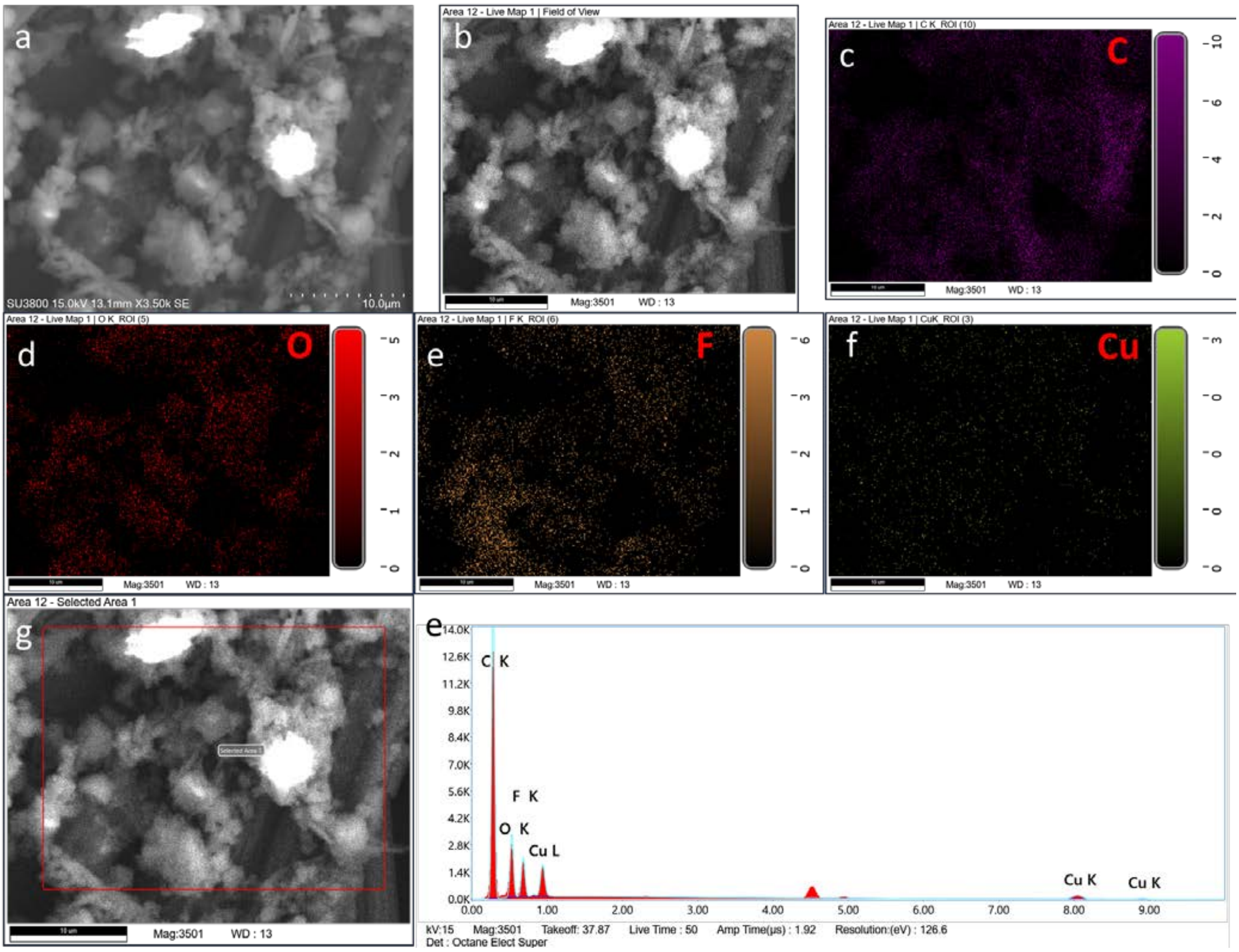

Figure S25. The SEM images (a), mapping (b-f), EDS (g, e) of Cu-SMS on carbon paper before test.

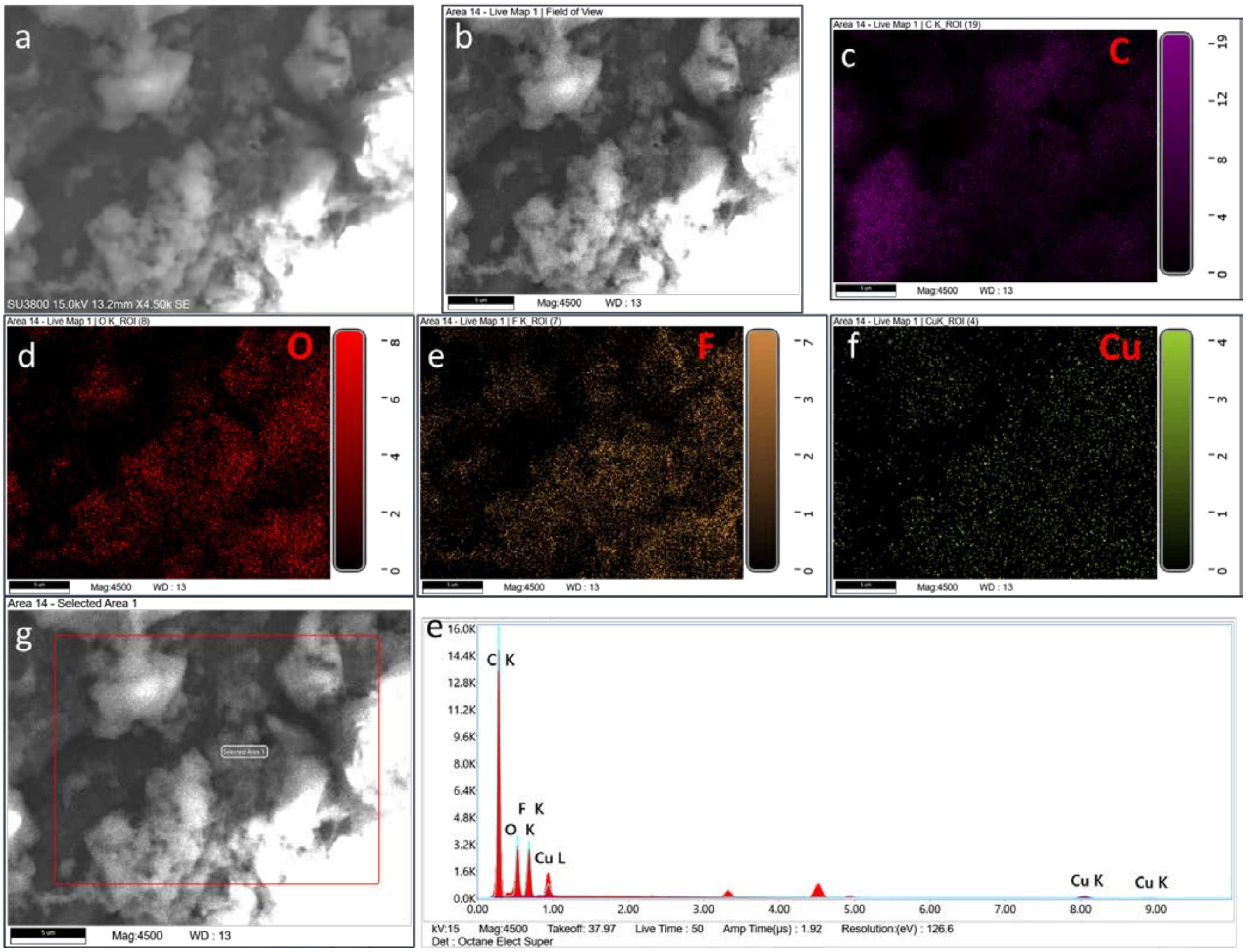

Figure S26. The SEM images (a), mapping (b-f), EDS (g, e) of Cu-SMS on carbon paper after test.

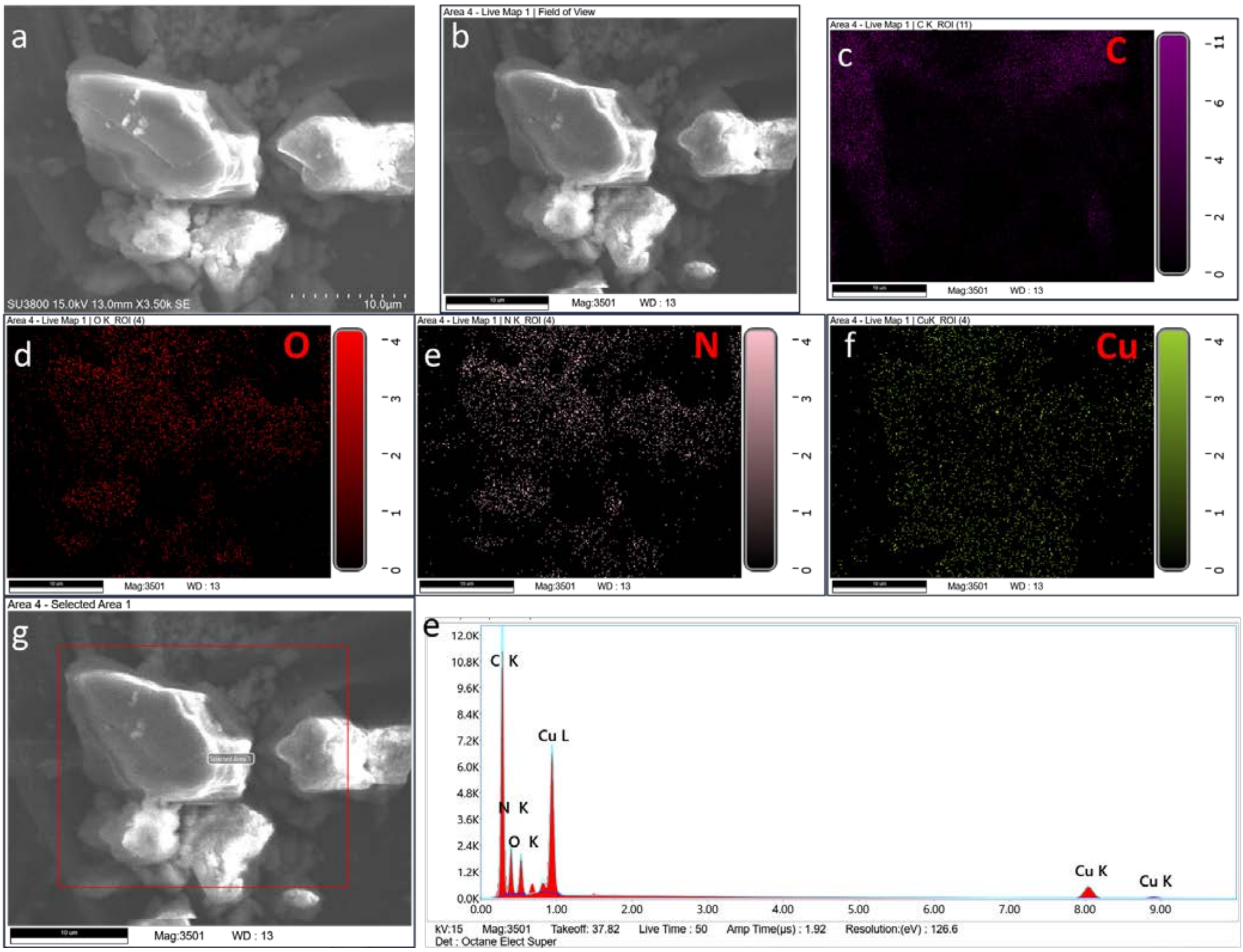

Figure S27. The SEM images (a), mapping (b-f), EDS (g, e) of Cu-MMS on carbon paper before test.

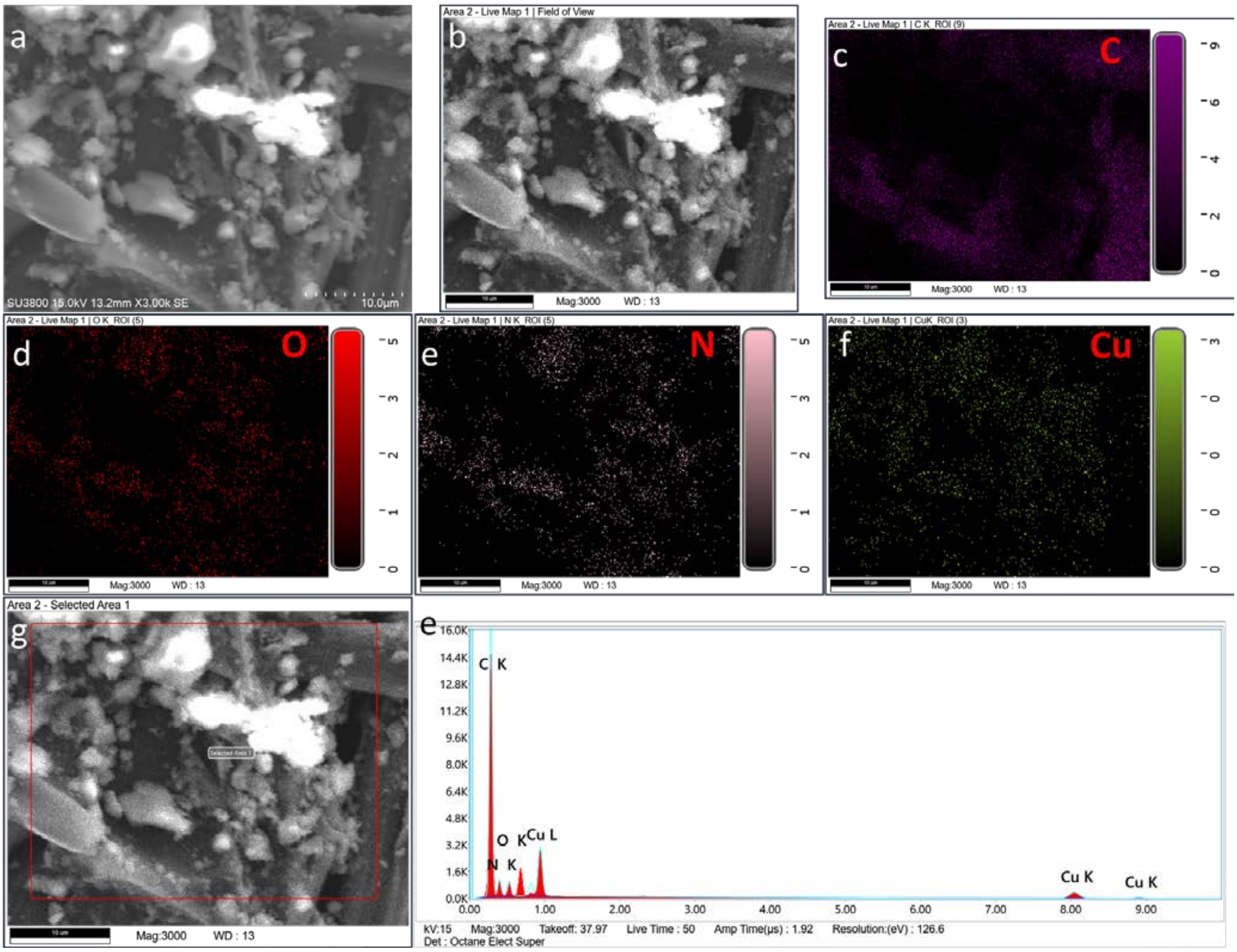

Figure S28. The SEM images (a), mapping (b-f), EDS (g, e) of Cu-MMS on carbon paper after test.

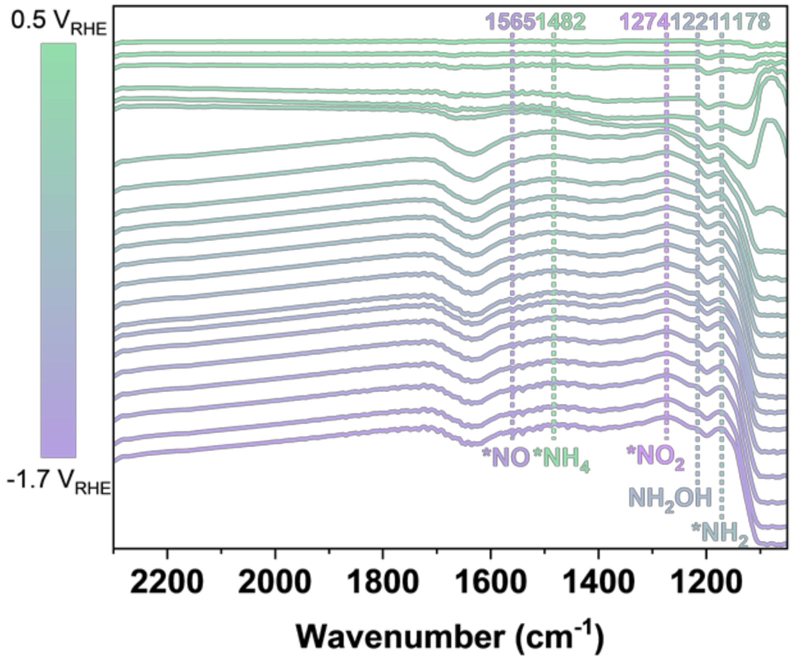

Figure S29. Potential-dependent in-situ ATR-SEIRAS on the Cu-SMS surfaces using $K^{14}NO_3$ solutions.

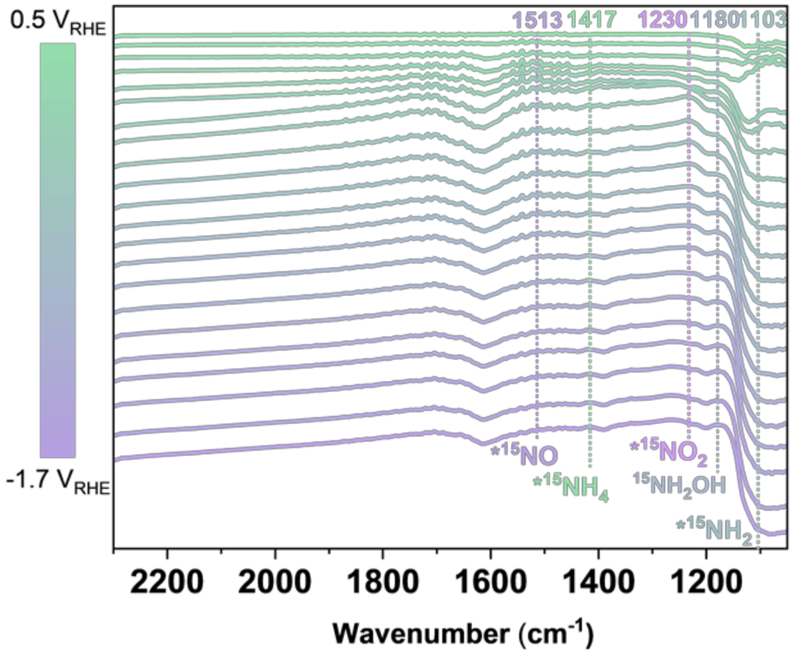

Figure S30. Potential-dependent in-situ ATR-SEIRAS on the Cu-SMS surfaces using $K^{15}NO_3$ solutions.

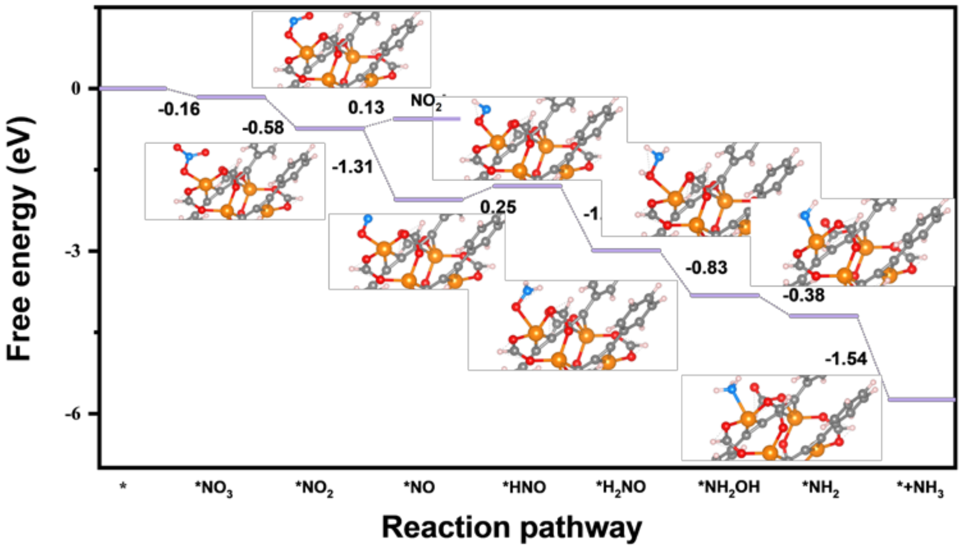

Figure S31. The Gibbs free energy diagrams for NO_3RR on Cu-SMS along the optimal pathway.

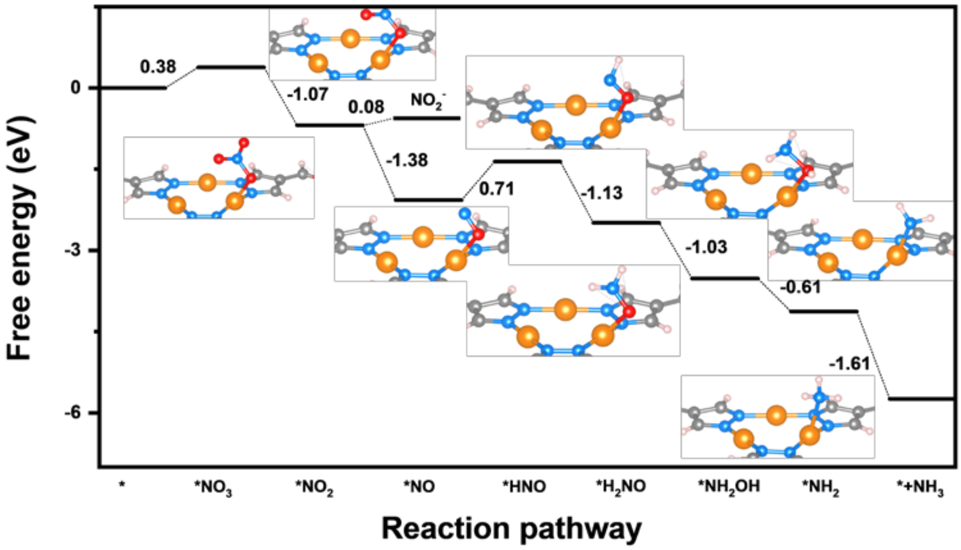

Figure S32. The Gibbs free energy diagrams for NO_3RR on Cu-MMS along the optimal pathway.

Table S1. Crystal data for Cu-SMS.

**Compounds**	**Cu-SMS**
CCDC	2403302
Empirical formula	$C_{40}H_{20}Cu_4F_{12}O_8$
Formula weight	1110.72
Temperature/K	149.98(10)
Crystal system	monoclinic
Space group	$P2_1/n$
a/Å	9.9855(2)
b/Å	8.7585(2)
c/Å	22.0889(4)
α/°	90
β/°	92.185(2)
γ/°	90
Volume/$Å^3$	1930.45(7)
Z	2
$\rho_{calc}g/cm^3$	1.911
μ/mm^{-1}	3.512
F(000)	1096.0
Radiation	Cu Kα (λ = 1.54184)
Index ranges	$-12 \leq h \leq 12$, $-6 \leq k \leq 10$, $-27 \leq l \leq 27$
Reflections collected	7000
Independent reflections	3743 [R_{int}= 0.0204, R_{sigma}= 0.0229]
Data/restraints/parameters	3743/0/289
Goodness-of-fit on F^2	1.097
Final R indexes [I>=2σ (I)]	R_1= 0.0348, wR_2= 0.0884
Final R indexes [all data]	R_1= 0.0369, wR_2= 0.0900
Largest diff. peak/hole/e $Å^{-3}$	0.41/-0.84

Table S2. Possible band assignments of in-situ ATR-SEIRAS.

Wavenumber (cm^{-1})	Assignment	Intermediate	References
~1565	N−O vibration	*NO	*Adv. Funct. Mater*., **2023**, 33, 2302651. [17]
~1482	N−H vibration	NH_4^+	*Adv. Funct. Mater*., **2023**, 33, 2209890. [18]<br>*Angew. Chem. Int. Ed*., **2023**, 62, e202300054. [19]<br>*Adv. Sci*. **2023**, 10, 2303789. [20]
~1274	N−O vibration	$*NO_2$	*Chem Catalysis*., **2023**, 3, 100595. [21]<br>*Nat. Cat*., **2023**, 6, 402-414. [22]<br>*Adv. Sci*., **2023**, 10, 2303789. [20]
~1221	N−H vibration	NH_2OH	*Chem Catalysis*., **2023**, 3, 100595. [21]
~1178	N−H vibration	$*NH_2$	*Adv. Funct. Mater*., **2023**, 33, 2302651. [17]<br>*Adv. Funct. Mater*., **2023**, 33, 2209890. [18]<br>*Nat. Cat*., **2023**, 6, 402-414. [22]<br>*J. Am. Chem. Soc*., **2024**, 146, 7779-7790. [23]

Table S3. The element content of the sample from −0.8 V to −1.4 V vs RHE based on the XPS spectrum.

	Atomic %			
Element	−0.8 V	−1.0 V	−1.2 V	−1.4 V
C 1s	41.1	42.8	47.4	37.9
F 1s	36.7	41.5	35.0	39.5
O 1s	13.1	9.2	10.6	12.8
Cu 2p	2.4	1.4	2.0	2.9

Table S4. The comparison of the NH_3 synthesis activity of Cu-SMS in NO_3RR with other catalysts under ambient conditions.

**Catalyst**	**Type**	**Operating potential**	**Product**	**FE (%)**	**Stability (XAS/XRD/UV-Vis/XPS)**	**Ref.**
Cu-SMS	Nanocluster	−1.40 V (RHE)	NH_3	>99	No restructuring (XRD/XAS/XPS/TEM)	This work
$Cu_2Cl_2(BIANP)_2$	Nanocluster	−0.946 V (RHE)	NH_3	94	No restructuring (XRD)	Angew. Chem. Int. Ed., 2024, e202413033. [24]
$Cu_2Br_2(BIANP)_2$	Nanocluster	−0.946 V (RHE)	NH_3	~78	No restructuring (XRD)	Angew. Chem. Int. Ed., 2024, e202413033. [24]
$Cu_2I_2(BIANP)_2$	Nanocluster	−1.046 V (RHE)	NH_3	~55	No restructuring (XRD)	Angew. Chem. Int. Ed., 2024, e202413033. [24]
Poly-Cu_{14}cba	Nanocluster	−1.05 V (RHE)	NH_3	90	No restructuring (XRD/XPS/TEM)	Angew. Chem. Int. Ed., 2022, 61, e202114538. [25]
Cu_{14}cba	Nanocluster	----	NH_3	50	----	Angew. Chem. Int. Ed., 2022, 61, e202114538. [25]
DiMe-Cu_3-MOF	MOF	−1.55 V (Ag/AgCl)	NH_3	95	No restructuring (XRD/TEM)	Chem. Sci., 2025, 16, 13503. [26]
UniMe-Cu_3-MOF	MOF	−1.55 V (Ag/AgCl)	NH_3	~60	No restructuring (XRD/TEM)	Chem. Sci., 2025, 16, 13503. [26]
UniMe-Cu_3-MOF	MOF	−1.65 V (Ag/AgCl)	NH_3	~58	No restructuring (XRD/TEM)	Chem. Sci., 2025, 16, 13503.[26]
Cu@Th-BPYDC	MOF	0 V (RHE)	NH_3	92.5	No restructuring (XRD)	ACS Cent. Sci.， 2021, 7, 1066−1072. [27]
CuO	Nanosheet	−0.70 V (RHE)	NH_3	97	Restructuring (XRD)	Mater. Chem. Front., 2025, 9, 2243. [28]

Cu	Nanoparticle	−1.25 V (RHE)	NH_3	85.4	Restructuring (STEM)	ACS Appl. Energy Mater. 2024, 7, 9, 3761–3775. [29]
Cu_1/WO_3	SAC	−0.60 V (RHE)	NH_3	93.7	No restructuring (XRD/XPS/TEM/HRTEM)	Angew. Chem. Int. Ed., 2025, 64, e202423154. [30]
p-CNCu^{s}La^n-m	SAC	−0.45 V (RHE)	NH_3	97.7	No restructuring (HRTEM)	Adv. Mater., 2025, 37, 2415632. [31]
HE Cu_1−N_4	SAC	−0.70 V (RHE)	NH_3	97.54	No restructuring (XRD)	Environ. Sci. Technol., 2025, 59, 8555−8567. [32]
Cu–N_1O_2	SAC	−0.60 V (RHE)	NH_3	96.5	----	Angew. Chem. Int. Ed., 2024, 63, e202409125. [33]
Cu–N_4B_2	SAC	−0.60 V (RHE)	NH_3	98.2	No restructuring (XPS)	Energy Environ. Sci., 2024, 17, 8360. [34]
Cu–N_3 SACs/NCNT	SAC	−0.80 V (RHE)	NH_3	89.64	Restructuring (XAS)	Adv. Funct. Mater., 2023, 33, 2302651. [17]
Cu–cis–N_2O_2	SAC	----	NH_3	88.46	----	Adv. Mater. 2022, 34, 2205767. [35]
Cu–N_4	SAC	−1.00 V (RHE)	NH_3	84.7	Restructuring (XAS)	J. Am. Chem. Soc., 2022, 144, 12062−12071. [36]

References

1. Sheldrick GMS. Program for area detector adsorption correction. *Institute for Inorganic Chemistry, University of Göttingen, Göttingen (Germany)*. 1996.
2. Dolomanov OV, Bourhis LJ, Gildea RJ *et al.* OLEX2: a complete structure solution, refinement and analysis program. *Journal of Applied Crystallography* 2009; **42**: 339–341.
3. Sheldrick GM. SHELXT-integrated space-group and crystal-structure determination. *Acta Crystallogr A Found Adv* 2015; **71**: 3–8.
4. Schlesiger C, Praetz S, Gnewkow R *et al.* Recent progress in the performance of HAPG based laboratory EXAFS and XANES spectrometers. *J Anal At Spectrom* 2020; **35**: 2298–2304.
5. Grigorieva I, Antonov A, Gudi G. Graphite optics—current opportunities, properties and limits. *Condensed Matter*. 2019; **4**: 18.
6. Dinapoli R, Bergamaschi A, Henrich B *et al.* EIGER: Next generation single photon counting detector for X-ray applications. *Nuclear Instruments and Methods in Physics Research Section A: Accelerators, Spectrometers, Detectors and Associated Equipment* 2011; **650**: 79–83.
7. Schlesiger C, Anklamm L, Stiel H *et al.* XAFS spectroscopy by an X-ray tube based spectrometer using a novel type of HOPG mosaic crystal and optimized image processing. *J Anal At Spectrom* 2015; **30**: 1080–1085.
8. Ravel B, Newville M. ATHENA, ARTEMIS, HEPHAESTUS: data analysis for X-ray absorption spectroscopy using IFEFFIT. *J Synchrotron Rad* 2005; **12**: 537–541.
9. G. Kresse av JF. Efficiency of ab-initio total energy calculations for metals and semiconductors using a plane-wave basis set. *Computational Materials Science* 1996; **6**: 15–50.
10. G.Kresse JF. Efficient iterative schemes for ab initio total-energy calculations using a plane-wave basis set. *Phys Rev B* 1996; **54**: 11169–11189.
11. J.P. Perdew KB, M. Ernzerhof. Generalized gradient approximation made simple. *Phys Rev Lett* 1996; **77**: 3865–3868.
12. Monkhorst HJ, Pack JD. Special points for brillouin-zone integrations. *Phys Rev B* 1976; **13**: 5188–5192.
13. Blochl PE. Projector augmented-wave method. *Phys Rev B* 1994; **50**: 17953–17979.
14. Grimme S, Antony J, Ehrlich S *et al.* A consistent and accurate ab initio parametrization of density functional dispersion correction (DFT-D) for the 94 elements H-Pu. *J Chem Phys* 2010; **132**: 154104.
15. Rossmeisl J, Logadottir A, Nørskov JK. Electrolysis of water on (oxidized) metal surfaces. *Chemical Physics* 2005; **319**: 178–184.
16. Peterson AA, Abild-Pedersen F, Studt F *et al.* How copper catalyzes the electroreduction of carbon dioxide into hydrocarbon fuels. *Energy Environ Sci* 2010; **3**: 1311–1315.
17. Wang Y, Zhang W, Wen W *et al.* Atomically dispersed unsaturated Cu–N_3 sites on high-curvature hierarchically porous carbon nanotube for synergetic enhanced nitrate electroreduction to ammonia. *Adv Funct Mater* 2023; **33**: 2302651.
18. Chen K, Ma Z, Li X *et al.* Single-atom Bi alloyed Pd metallene for nitrate electroreduction to ammonia. *Adv Funct Mater* 2023; **33**: 2209890.
19. Zhang G, Li X, Chen K *et al.* Tandem electrocatalytic nitrate reduction to ammonia on MBenes. *Angew Chem Int Ed* 2023; **62**: e202300054.

20. Wu S, Jiang Y, Luo W *et al.* Ag-Co_3O_4-CoOOH-nanowires tandem catalyst for efficient electrocatalytic conversion of nitrate to ammonia at low overpotential via triple reactions. *Adv Sci* 2023; **10**: 2303789.
21. Guiet A, Simonin A, Bemana H *et al.* Reversible transition of an amorphous Cu-Al oxyfluoride into a highly active electrocatalyst for NO_3^- reduction to NH_3. *Chem Catal* 2023; **3**: 100595.
22. Han S, Li H, Li T *et al.* Ultralow overpotential nitrate reduction to ammonia via a three-step relay mechanism. *Nat Catal* 2023; **6**: 402–414.
23. Liu K, Li H, Xie M *et al.* Thermally enhanced relay electrocatalysis of nitrate-to-ammonia reduction over single-atom-alloy oxides. *J Am Chem Soc* 2024; **146**: 7779–7790.
24. Zheng SJ, Dong XY, Chen H *et al.* Unveiling ionized interfacial water-induced localized H^* enrichment for electrocatalytic nitrate reduction. *Angew Chem Int Ed* 2024; **64**: e202413033.
25. Wang YM, Cai J, Wang QY *et al.* Electropolymerization of metal clusters establishing a versatile platform for enhanced catalysis performance. *Angew Chem Int Ed* 2022; **61**: e202114538.
26. Fu XX, Guo H, Si DH *et al.* Hydrogen-bond mediated electrocatalytic nitrate reduction to ammonia over metal-organic frameworks with industrial current density. *Chem Sci* 2025; **16**: 13503–13513.
27. Gao Z, Lai Y, Tao Y *et al.* Constructing well-defined and robust Th-MOF-supported single-site copper for production and storage of ammonia from electroreduction of nitrate. *ACS Cent Sci* 2021; **7**: 1066–1072.
28. Thani E-S, Yang Y-T, Du Q-Y *et al.* Porous Cu nanosheets for efficient ammonia production via nitrate electroreduction. *Mater Chem Front* 2025; **9**: 2243–2249.
29. Hoekx S, Daems N, Arenas Esteban D *et al.* Toward the rational design of Cu electrocatalysts for improved performance of the NO_3RR. *ACS Appl Energy Mater* 2024; **7**: 3761–3775.
30. Shen F, He S, Tang X *et al.* Breaking linear scaling relation limitations on a dual-driven single-atom copper/tungsten oxide catalyst for ammonia synthesis. *Angew Chem Int Ed* 2025; **64**: e202423154.
31. Zuo Y, Sun M, Li T *et al.* Capturing copper single atom in proton donor stimulated O-end nitrate reduction. *Adv Mater* 2025; **37**: e2415632.
32. Long X, Huang F, Zhong T *et al.* One-step strategy to maximize single-atom catalyst utilization in nitrate reduction via bidirectional optimization of mass transfer and electron supply. *Environ Sci Technol* 2025; **59**: 8555–8567.
33. Gu Z, Zhang Y, Fu Y *et al.* Coordination desymmetrization of copper single-atom catalyst for efficient nitrate reduction. *Angew Chem Int Ed* 2024; **63**: e202409125.
34. Huang T, Liang T, You J *et al.* Coordination environment-tailored electronic structure of single atomic copper sites for efficient electrochemical nitrate reduction toward ammonia. *Energy Environ Sci* 2024; **17**: 8360–8367.
35. Cheng XF, He JH, Ji HQ *et al.* Coordination symmetry breaking of single-atom catalysts for robust and efficient nitrate electroreduction to ammonia. *Adv Mater* 2022; **34**: e2205767.
36. Yang J, Qi H, Li A *et al.* Potential-driven restructuring of Cu single atoms to nanoparticles for boosting the electrochemical reduction of nitrate to ammonia. *J Am Chem Soc* 2022; **144**: 12062–12071.
